# Unraveling the complexity of drug resistance mechanisms to SINE, T cell-engaging therapies and CELMoDs in multiple myeloma: a comprehensive review

**DOI:** 10.20517/cdr.2024.39

**Published:** 2024-06-26

**Authors:** Jacqueline Schütt, Kerstin Brinkert, Andrzej Plis, Tino Schenk, Annamaria Brioli

**Affiliations:** ^1^Clinic for Hematology, Hemostasis, Oncology and Stem cell transplantation, Hannover Medical School, Hannover 30625, Germany.; ^2^Clinic for Internal Medicine C, Hematology and Oncology, Greifswald University Medicine, Greifswald 17489, Germany.; ^3^Clinic of Internal Medicine 2, Department of Hematology and Medical Oncology, Jena University Hospital, Jena 07741, Germany.; ^4^Institute of Molecular Cell Biology, CMB, Jena University Hospital, Jena 07741, Germany.; ^#^Authors contributed equally.

**Keywords:** Multiple myeloma, SINE, selinexor, CELMoD, bispecific antibodies, CAR-T cells, mechanism of resistance

## Abstract

Despite significant advances in the understanding of multiple myeloma (MM) biology and the development of novel treatment strategies in the last two decades, MM is still an incurable disease. Novel drugs with alternative mechanisms of action, such as selective inhibitors of nuclear export (SINE), modulators of the ubiquitin pathway [cereblon E3 ligase modulatory drugs (CELMoDs)], and T cell redirecting (TCR) therapy, have led to significant improvement in patient outcomes. However, resistance still emerges, posing a major problem for the treatment of myeloma patients. This review summarizes current data on treatment with SINE, TCR therapy, and CELMoDs and explores their mechanism of resistance. Understanding these resistance mechanisms is critical for developing strategies to overcome treatment failure and improve therapeutic outcomes.

## INTRODUCTION

Multiple myeloma (MM) is the second most frequent hematologic malignancy, with an incidence of 8/100,000 persons in Europe^[1]^. Despite significant improvement in MM therapy, no treatment so far has been able to cure MM patients. The introduction of novel agents such as proteasome inhibitors (PIs), immunomodulatory drugs (IMiDs) and cereblon E3 ligase modulatory drugs (CELMoDs), selective inhibitors of nuclear export (SINE), monoclonal antibodies (MoAbs), T cell engagers (bispecific antibodies), and chimeric antigen receptor (CAR)-T cell therapy has revolutionized MM treatment^[2-8]^. Nevertheless, relapse will inevitably arise, and the emergence of drug resistance represents a significant obstacle to achieving durable responses due to the development of multi-resistant disease. In the absence of novel agents, triple refractory patients (i.e., patients that have become resistant to a PI, an IMiD and a MoAb) have a dismal prognosis with a survival of approximately 4 to 12 months^[9,10]^. Compounds such as CAR-T cells or bispecific antibodies have shown remarkable efficacy both in triple-class exposed MM patients^[2-5]^ and in earlier lines of therapy^[11,12]^, with unprecedented rates of responses and progression-free survival (PFS) benefits. Still, PFS curves do not plateau, clearly pointing out the pivotal importance of understanding the mechanisms of resistance and how to overcome them. Over the past decades, extensive research efforts have been made to shed light on the multifaceted nature of drug resistance in MM, revealing a complex interplay of tumor cell-intrinsic factors, microenvironmental influences, and treatment-induced adaptations.

Microenvironmental factors play a pivotal role in promoting drug resistance^[13]^. Bone marrow stromal cells secrete several cytokines [such as interleukin 6 (IL6), transforming growth factor beta (TGF-β), and insulin-like growth factor 1 (IGF-1)] that promote plasma cell growth and survival^[14,15]^. The interaction between plasma cells, osteoblasts, and osteoclasts not only fosters plasma cell growth but is also responsible for the development of bone disease, one of the most frequent complications of MM patients^[16-18]^. Despite MM cells inducing neo-angiogenesis^[19]^, the bone marrow microenvironment is a hypoxic environment. Hypoxia modifies the metabolism of MM cells, and this has been linked to epigenetic deregulation and the development of treatment resistance^[20-22]^. With the development of immunotherapies for the treatment of cancer in general, and myeloma in particular, interest in the contribution of the immune microenvironment to disease resistance has increased. Changes in the immune system occur early in the development of myeloma^[23]^, and the bone marrow niche is an immunosuppressive environment characterized by increased activity of myeloid-derived suppressor cells (MDSC). MDSC are supported by IL6 that is secreted by MM cells. MDSC not only inhibit T cell function, but are also able to stimulate angiogenesis via vascular endothelial growth factor (VEGF) secretion in a feed-forward loop that is beneficial for MM growth and survival^[24-26]^.

Despite the advances made in understanding the development of treatment resistance, resistance mechanisms to the most recently approved therapies or to therapies in advanced phase clinical trials but not yet approved are still poorly explored and understood. Although much research has been performed on resistance to IMiDs, the specific mechanisms of resistance to CELMoDs have only been partially elucidated. Even more complicated is the situation for SINE, where the mechanisms of resistance are still largely unknown. An exception in this respect is T cell redirecting (TCR) therapies, such as bispecific antibodies and CAR-T cells, where the extensive research being conducted has identified different potential resistance mechanisms^[27]^. Nevertheless, the underlying reasons why some patients will respond to TCR therapies, and some will not, are still not completely understood and no predictive marker has been validated so far to identify those patients who will profit the most (or the least) from these therapies.

By synthesizing the latest research findings and clinical insights, this comprehensive review aims to provide a thorough exploration of the diverse mechanisms driving drug resistance in MM, focusing on the most recent therapies, such as SINE, TCR therapy (bispecific antibodies and CAR-T), and CELMoDs.

## SINE

SINE are a class of drugs that work specifically by blocking the export of tumor suppressors, growth-regulatory proteins and RNA from the nucleus into the cytoplasm, thereby interfering with normal cellular functions. Trafficking of RNA, ribosomes, and proteins (such as tumor suppressor proteins, cell cycle inhibitors, and transcription regulators) between the nucleus and the cytoplasm is highly important for cell function. Alterations in this process, influencing the quantity of protein released into the cytoplasm or retained in the nucleus, can be the starting point of different pathological processes, including the development of malignancies. The export of RNA and proteins from the nucleus to the cytoplasm is regulated by the nuclear pore complex (NCP), together with transport receptor molecules such as exportins. Exportins, a ubiquitous protein family, utilize the nuclear export signal (NES) of cargo proteins to facilitate their transport out of the nucleus. This makes exportins valuable targets for SINE compounds^[28]^. One of the most studied and targetable exportins is nuclear export protein 1 (XPO1), also known as chromosome region maintenance 1 (CRM1)^[29]^. For the cargo protein to bind to the XPO1, a leucine-rich NES must be present and accessible to XPO1 on the cargo^[30]^. Different modifications in the cargo protein, such as phosphorylation, dephosphorylation, acetylation, sumoylation, and ubiquitination, are responsible for the accessibility of this NES domain^[31,32]^. Overexpression of XPO1 has been observed in several cancers, including MM, and correlates with shorter event-free survival (EFS) and shorter overall survival (OS)^[33-36]^. XPO1 regulates the nuclear export of mRNA transcripts and of more than 200 proteins, including oncogenic proteins and tumor suppressor proteins^[37]^. Particularly interesting for cancer development is the regulation of the transport of p53, APC/β-catenin, FOXO3, BRCA 1/2, IkBa, surviving, c-MYC, and BCR::ABL1^[38,39]^. For example, if p53, a major tumor suppressor protein involved in different cancers, is exported outside of the nucleus, it loses its antitumor effects, which are retained when p53 remains in the nucleus^[40-42]^. Similar is the situation for BRCA1, an important driver of breast cancer. Inhibition of XPO1 induces accumulation of BRCA1 in the nucleus, whereas overexpression of XPO1 has the opposite effect^[43]^. Retention of BCR::ABL1 into the nucleus is able to induce apoptosis, making SINE interesting compounds for patients with chronic myeloid leukemia (CML)^[38]^. Different is the effect of SINE on oncogenes. XPO1 regulates the nuclear export of mRNA encoding oncoproteins such as MYC. Inhibition of XPO1 downregulates MYC expression in several tumors and has been suggested as an effective therapeutic strategy in double-hit lymphomas^[44-48]^. SINE compounds, inhibiting XPO1, disrupt nuclear-cytoplasmic shuttling, causing the accumulation of proteins and mRNA in the nucleus. This effect ultimately leads to a reduction in oncoproteins, the nuclear retention of tumor suppressor proteins, and the induction of apoptosis. Topoisomerases can also be affected by XPO1, resulting in reduced efficacy of topoisomerase inhibitor drugs such as anthracyclines and etoposide^[29]^. Topoisomerases are essential for cell division and are involved in DNA replication, transcription, and modification of chromatin conformation^[49]^. Inhibiting topoisomerases with, e.g., anthracyclines or etoposide, results in double-strand DNA breaks and cell death. For this effect to occur, topoisomerases must be localized in the nucleus. XPO1 exports topoisomerases from the nucleus to the cytoplasm^[50]^, and this effect is stronger when myeloma cells are present at high density^[51]^. Inhibiting XPO1 by blocking topoisomerases in the nucleus can re-sensitize myeloma cells to the effects of anthracyclines and etoposide^[29,52]^.

Importantly, SINE can also re-sensitize resistant cells to conventional drugs and PIs^[52-55]^. In MM, SINE compounds not only exert their anticancer effect by acting directly on MM cells, but also interfere with the tumor microenvironment. SINE inhibit receptor activator of nuclear factor kappa-Β ligand (RANKL)-induced NFκB activity and nuclear factor of activated T cells 1 (NFATc1), regulating osteoclast differentiation. Blockage of NFκB and NFATc1 results in impaired osteoclast differentiation, suggesting that SINE might have a role in preventing MM-related bone disease^[46]^.

Selinexor, a specific inhibitor of XPO1, is the first Food and Drug Administration (FDA)-approved inhibitor of nuclear export and was granted accelerated approval in July 2019. In Europe, it was approved in January 2021. Approval was based on the data of the STORM trial (NCT02336815), a phase 2b, international, multicenter, open-label study conducted in the USA and Europe^[6]^. The trial population was heavily pretreated, as inclusion criteria demanded that patients had been previously treated with two PIs, two IMiDs, monoclonal antibodies, and alkylating agents. Additionally, patients had to be progressing under their last line of therapy. In this difficult-to-treat population, an overall response rate (ORR) [defined as partial response (PR) or better] was achieved in 26% of patients. Median PFS and OS were 4 and 9 months, respectively^[6]^.

Due to the promising data of selinexor combined with dexamethasone, the phase 1/2 STOMP trial was started. This trial aims at exploring different combinations and schedules of selinexor and is designed as an open-label, 12-arm, parallel-group study enrolling both patients with relapsed and/or refractory MM (RRMM) as well as those with newly diagnosed MM (NDMM). Combinations explored include pomalidomide, lenalidomide, carfilzomib, bortezomib, and daratumumab. Responses were encouraging in the PI arms, with an ORR for the selinexor/carfilzomib/dexamethasone (XKd) combination of 78% and an ORR for the selinexor/bortezomib/dexamethasone (XVd) arm of 63%^[56,57]^. Interestingly, in the bortezomib arm, PI-refractory patients showed an ORR of 43%, confirming preclinical data^[52,55]^ that inhibition of XPO1 might re-sensitize MM cells to proteasome inhibition^[57]^. These data set the basis for the phase 3 Boston trial (NCT03110562), comparing XVd with Vd. Patients who had received one to three prior anti-MM regimens were randomized 1:1 to receive XVd or Vd. Treatment was continued until disease progression. The trial met its primary endpoint, with a statistically significant increase in median PFS. Median PFS was 14 months in the XVd arm compared with 10 months in the Vd arm [hazard ratio (HR) 0.70; *P* = 0.0075]. ORR was 76% and 62%, respectively, with 17% of patients in the XVd arm achieving at least a complete response (CR) *vs.* 10% in the Vd arm^[58]^. These data led to the FDA approval of the combination XVd from the second line of therapy in December 2020 and to the European Medicines Agency (EMA) approval in July 2022.

### SINE mechanism of resistance

Despite encouraging trial data, resistance to selinexor does occur, and patients will inevitably relapse. The mechanism of resistance to selinexor in MM has been poorly explored so far, as the majority of research has been focused on how selinexor and other SINE inhibitors can reduce resistance to other drugs^[52-55]^. Looking at different cancers, it is clear that resistance mechanisms to SINE are pleiotropic and can vary according to the type of neoplasia.

Mutations of XPO1 seem to play a marginal role in the development of SINE resistance. The *in vitro* production of selinexor-resistant fibrosarcoma cell lines did not reveal the emergence of mutations, suggesting that in fibrosarcoma, resistance to XPO1 inhibition is not due to mutation of the target which could prevent the binding of the drug^[59]^. Analysis of primary mediastinal B cell lymphoma cell lines showed recurrent mutations (E517K and E517G) in the NES-binding groove. However, the presence of E571 mutations did not affect response to selinexor therapy^[60]^. Mutations of E517 are present in 5% of chronic lymphocytic leukemia (CLL) patients. The presence of the E571 mutations increased CLL aggressiveness in *in vivo* models, but did not affect selinexor binding to XPO1^[61]^, suggesting that mutation of the target, by not altering the binding of SINE to XPO1, has a marginal role in SINE resistance. The only mutation so far that has been able to induce resistance to SINE is C528S, a mutation engineered in the lab to specifically affect selinexor binding site^[62]^. Heterozygous C528S is sufficient to induce selinexor resistance, indicating that a single mutation of cysteine_528_ can cause resistance to selinexor^[63]^.

Gene expression profiling of sensitive and resistant fibrosarcoma cell lines showed genetic changes in the same direction after treatment with the SINE compound KPT-185. These data suggest that resistance to SINE compounds is likely not to be caused by a single resistance mechanism, but rather by a reduced sensitivity to the overall XPO1 inhibition, although mechanism leading to this reduced sensitivity remain elusive^[59]^.

Further analysis of the resistance mechanism has therefore focused on altered pathways. Upregulation of the NFκB pathway was observed in SINE-resistant fibrosarcoma cell lines. The importance of upregulation of NFκB in resistance to SINE compounds was also confirmed in osteosarcoma cell lines, providing a rationale for combining SINE with PIs to overcome resistance^[64]^. This is particularly important, as PIs form the backbone of many MM therapies.

In ovarian cancers, increase of the neuregulin 1 (NRG1)/Erb-B2 receptor tyrosine kinase 3 (ERBB3) pathway has been found to contribute to SINE resistance. Expression of NRG1 and ERBB3, as well as NRG1 secretion, were increased in SINE-resistant ovarian cancer cells. Downregulation of ERBB3 restored SINE sensitivity, while cells cultivated in the presence of exogenous NRG1 showed reduced sensitivity to KPT-185^[65]^.

In CML cell lines, resistance to SINE has been postulated to be linked to reduced ferroptosis. A single-cell dynamic transcriptomic analysis of the CML cell line K562 (parental and selinexor-resistant) found that ferroptosis-inhibitory molecules FTH1 and SLC7A11 were increased in selinexor-resistant K562 cells. This increased expression correlated with increased drug resistance. On the other hand, the expression of HMGB1 and MTDH, two ferroptosis-driving molecules, was decreased in resistant cell lines. Supporting these data, the ferroptosis inducer RSL3 was able to restore cell sensitivity to selinexor (also known as KPT-330)^[66]^.

In MM, the first efforts to identify the mechanism of resistance or response to selinexor were performed in the STORM trial. RNA sequencing of 32 patients revealed overexpression of E2F1 to be significantly related to a PFS shorter than 120 days. E2F1 is part of a family of transcription factors that regulate cell cycle progression, and its export from the nucleus to the cytoplasm is regulated by XPO1. The authors postulated that overexpression of E2F1 may result in downstream gene programming that confers a proliferative advantage in myeloma cells, and that E2F1 overexpression can be used as a marker of selinexor resistance^[67]^. Additionally, in patients enrolled in the STORM trial, a signature of four proteins (IRF3, ARL2BP, ZBTB17, and ATRX) was found to discriminate responders from non-responders^[6]^. A major limitation of this signature, however, is that it was developed only in 35 patients and validated in 12 patients. To further unravel the mechanism of selinexor resistance, Lagana *et al.* analyzed the transcriptome of 54 patients treated in the STORM trial using a machine learning approach. They identified three groups of patients with different PFS; patients with the poorest prognosis were characterized by upregulation of melanoma antigen family A (MAGE-A)^[68]^. MAGE-A is aberrantly expressed in MM, and can foster resistance through the downregulation of Bcl-2 interacting mediator of cell death (BIM) and p21^Cip1^^[69,70]^. MAGE-A-depleted MM cell lines NCI-H929 and RPMI8226 increased their sensitivity to selinexor compared to patients in whom MAGE-A was not depleted, confirming the role of MAGE-A in selinexor resistance^[68]^.

Recently, the same group refined their model on 256 selinexor-treated MM patients, identifying a three-gene signature capable of predicting response to selinexor. Upregulation of WNT10A, DUSP1, and ETCV7 correlated with longer PFS and a deeper response in patients treated in the Boston trial as well as in those treated with selinexor outside clinical trials. Despite showing a linear association with PFS, the signature did not reach statistical significance in patients treated in the STORM trial. Interestingly, the signature was not predictive of survival in MM patients who did not receive selinexor, but retained its predictive value in patients with glioblastoma treated with selinexor therapy (trend toward improved PFS and significantly higher rate of PR or better for patients with a higher signature expression)^[71]^. These data suggest that this signature is not disease-specific but is associated with sensitivity or resistance to selinexor independently of cancer type.

Using single-cell RNA sequencing on 21 patients treated with selinexor combination in the XPORT-MM-028 trial, Cohen *et al.* showed upregulation of XPOT and KPNB1 in selinexor refractory patients. XPOT is a tRNA transport, and *KPNB1* codifies for a nucleocytoplasmic transporter. Aligning with this, among the upregulated pathways in selinexor-refractory patients, they found mRNA splicing and capping as well as nucleocytoplasmic transport. These data suggest that alternative nuclear export pathways are another potential mechanism that can circumvent reduced nuclear transport mediated by XPO1 inhibition^[72]^.

Recently, heterogeneous nuclear ribonucleoprotein U (HNRNPU) has been found to regulate response to selinexor^[73]^. HNRNPU is a component of hnRNP complexes. hnRNPs are nuclear RNA-binging proteins that form complexes with RNA polymerase II transcripts. hnRNPs are involved in RNA metabolism, ranging from RNA transcription and pre-mRNA processing in the nucleus to translation and turnover of cytoplasmatic mRNA^[74]^. HNRNPU and XPO1 are strongly co-expressed in MM cells. HNRNPU affects XPO1-mediated nuclear export of ribosome subunits by affecting the localization of LTV1 and NMD3, two proteins involved in ribosome complex exportation from the nucleus to the cytosol. In cases with low HNRNPU, LTV1 and NMD3 are retained in the nucleus, reducing ribosome activity in the cytosol. This is important as ribosome nucleo-cytoplasmic transportation is linked to selinexor sensitivity^[75]^. HNRNPU also binds to the mRNA of MDM2 and RAN, altering their translation activity. Knockdown of HNRNPU increased selinexor sensitivity both *in vitro* and *in vivo*. The importance of HNRNPU in mediating selinexor resistance was confirmed by the fact that patients with a low HNRNPU expression had a better response to selinexor^[73]^.

Further work has identified overexpression of ABCC4 as a marker of response to selinexor, whereas reduced levels are associated with decreased response to selinexor^[76]^. Opposite to pathways inducing selinexor resistance, mechanisms inducing selinexor sensitivity have also been described. The knockdown of eIF4A was able to sensitize MM cells to selinexor, suggesting that a combination of selinexor with inhibitors of eIF4A could overcome treatment resistance^[77]^.

Another study found enrichment for genes involved in upregulated interferon signaling in patients responding to selinexor in combination with bortezomib and dexamethasone^[71]^. As interferon has been shown to modulate response to XPO1 inhibition^[78]^, it can be postulated that upregulation of interferon-mediated apoptotic signaling might prime cells to selinexor therapy.

**Figure 1:**
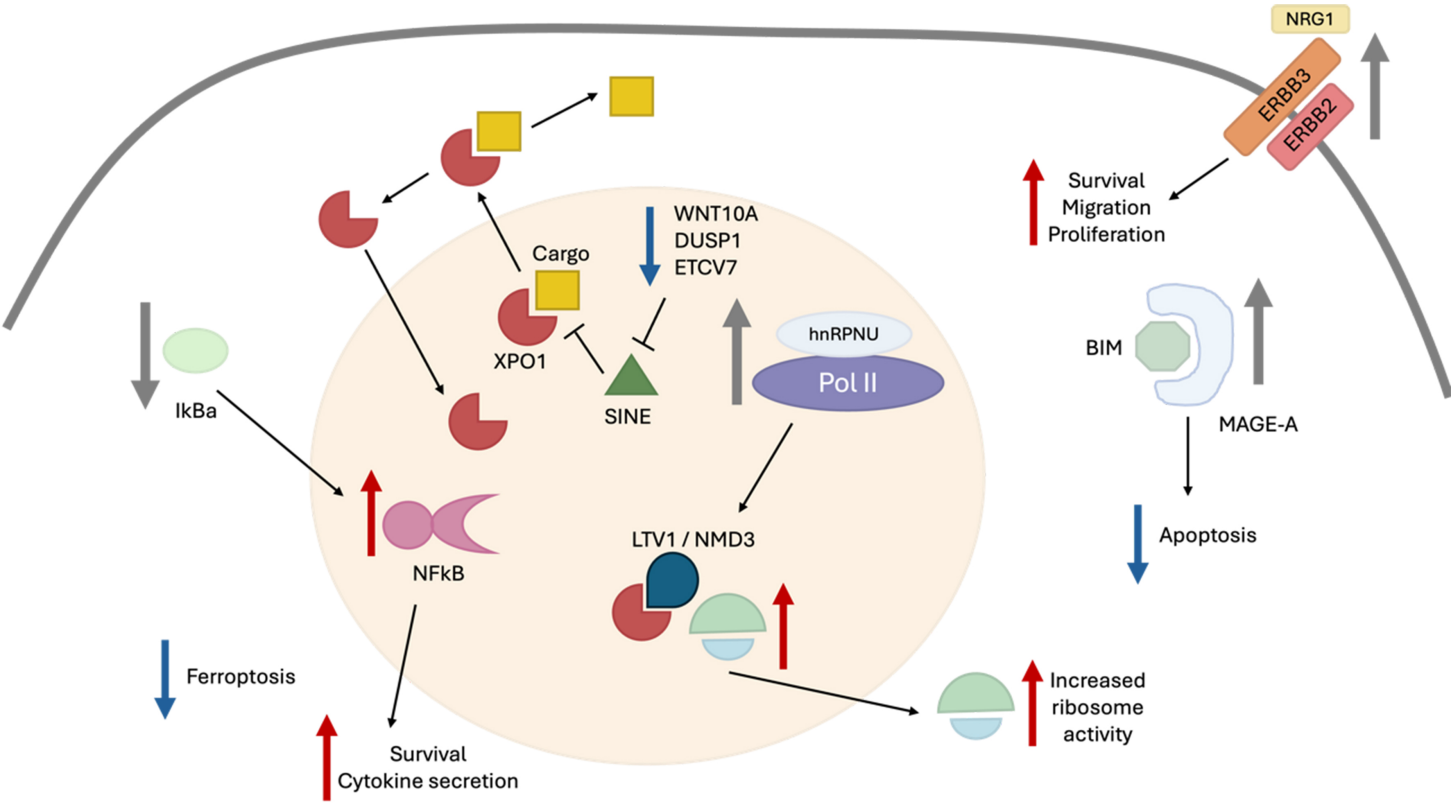
Schematic representation of the main mechanism of resistance to SINE compounds. Increased expression of NRG1 and ERBB3 contributes to SINE resistance by increasing cell survival, migration, and proliferation. Increased expression of MAGE-A reduces apoptosis, fostering resistance to SINE. Downregulation of WNT10A, DUSP1, and ETCV7 reduces SINE effectiveness. Reduction in ferroptosis also contributes to SINE resistance. Increased expression of HNRNPU, by modulating ribosome activity, increases resistance to SINE. For detailed explanations of SINE mechanisms of resistance, see the main text. SINE: Selective inhibitors of nuclear export; NRG1: neuroregulin 1; ERBB3: Erb-B2 receptor tyrosine kinase 3; MAGE-A: melanoma antigen family A; HNRNPU: heterogeneous nuclear ribonucleoprotein U; IkBa: nuclear factor of kappa light polypeptide gene enhancer in B-cells inhibitor alpha; NFκB: nuclear factor kappa-B; XPO1: exportin 1; hnRNPU: heterogeneous nuclear ribonucleprotein U; Pol II: RNA polymerase II; BIM: Bcl-2 interacting mediator of cell death.

Figure 1 illustrates the main mechanisms of resistance to SINE.

## TCR THERAPY

One of the greatest improvements in MM therapy has been made with the introduction of TCR therapy. The main effect of TCR therapy, such as bispecific antibodies or CAR-T cells, is to activate the host T cells to be able to specifically recognize and kill tumor cells. Bispecific antibodies such as teclistamab, elranatamab and talquetamab and the CAR-T cell products idecabtagene vicleucel (ide-cel) and ciltacabtagene autoleucel (cilta-cel) have dramatically changed the prognosis of triple-class refractory myeloma patients^[2-5,8]^. Bispecific antibodies bring the host’s T cell in contact with the malignant plasma cells by binding a surface antigen of choice on the plasma cells [typically B cell maturation antigen (BCMA) or G protein-coupled receptor family C group 5 member D (GPRC5D)] and the T cell receptor of the T cells. Through this binding, T cells are activated and can induce myeloma cell killing^[79,80]^. Most bispecific antibodies used in the clinic nowadays contain an Fc region, which prolongs the half-life of the antibody, reducing the need for frequent dosing. CAR-T cells are T cells that have been modified *ex vivo* to contain a CAR that can be activated on T cells by direct antigen contact without the need for major histocompatibility complex (MHC) class I molecules. This receptor consists of different parts: extracellularly, there is a binding domain for the antigen of interest, typically derived from a monoclonal antibody, where the heavy and light chains are linked to form a single chain variable fragment (scFv). The scFv is linked to a spacer (an Ig-like domain) and a transmembrane domain. In the new generation of CAR-Ts, the transmembrane domain is followed by one or two costimulatory domains, with the function of promoting CAR-T cell proliferation and survival. Finally, the intracellular moiety, containing the CD3ζ signaling chain of the T cell receptor, is responsible for T cell activation. This chimeric antigen produced *in vitro* is inserted *ex vivo* in patient T cells, which become able to recognize and kill myeloma cells even in the absence of MHC class I. The patient’s own CAR-T cells are then reinfused in the host after a lymphodepleting chemotherapy^[81]^. The use of bispecific antibodies and CAR-T cells in MM has been able to modify the course of the disease. Historical data on daratumumab refractory patients attested an OS of less than 1 year^[9]^, confirmed by the prospective observational LocoMMotion trial, which reported a PFS of 4.6 months (95%CI: 3.9-5.6) and an OS of 12.4 months (95%CI: 10.3-NE) in triple-class exposed patients^[10]^. In contrast, treatment with bispecific antibodies resulted in a PFS ranging from 12 to more than 15 months^[2,3,8]^, while treatment with CAR-T cells can lead to even better results with a PFS longer than 2 years^[4,82]^.

The first bispecific antibody to be approved was teclistamab. Teclistamab is a bispecific antibody that targets BCMA and was approved in August 2022 by EMA and in October 2022 by the FDA. Approval was based on the data on the MajesTEC-1 trial (NCT03145181), a phase 1/2 clinical trial for RRMM patients. The MajesTEC-1 trial enrolled patients who had undergone a median of 5 prior lines of therapy, with 76% classified as triple-class refractory. ORR was 63% and 39% of the patients achieved a CR or better; the median PFS was 11 months^[3]^.

The second approved bispecific antibody against BCMA is elranatamab. Elranatamb was approved in August 2023 by the FDA and in December 2023 by EMA. Authorization was based on data from cohort A of the phase 2 MagnetisMM-3 study (NCT04649359). The trial population was similar to the one enrolled in the MajesTEC-1 trial (median lines of prior therapy was 5), although a higher percentage of patients (97%) was triple-class refractory. The ORR was 61%, with 35% of patients achieving a CR or better. Fifteen-month rates for PFS and OS were 51% and 57%, respectively^[8]^.

Talquetamab is a bispecific antibody directed against GPRC5D. It was approved in August 2023 by the FDA and EMA based on the data of the phase 1/2 MonumenTAL-1 trial (NCT03399799). Patients enrolled in the trial received two different schedules of talquetamab, 405 µg/kg every week or 800 µg/kg every other week. Patients had a median of 6 prior lines of therapy and 75% were triple-class refractory. ORR was 70% in the 405 µg group and 64% in the 800 µg cohort. In both groups, 23% of patients achieved at least a CR. The median duration of response was 10 and 8 months in the 405 and 800 µg groups, respectively^[2]^.

Although CAR-T cells targeting GPRC5D are being developed and tested in clinical trials, at present, only CAR-T cells targeting BCMA have been approved in MM. The first CAR-T cell product to be available outside clinical trials was ide-cel, which was approved in March 2021 by the FDA and August 2021 by EMA. Approval was based on the data of the KarMMa trial (NCT03361748), a phase 1/2 trial investigating a single dose of ide-cel in RRMM patients. Patients enrolled in the trial had received a median of 6 prior lines of therapy and 84% were triple refractory. ORR was 73%, with 33% of patients achieving at least a CR. Of these, 79% were MRD-negative. Median progression-free survival was 9 months^[5]^. In a subsequent phase 3 clinical trial (NCT03651128), ide-cel confirmed its benefit upon the standard of care in triple-class exposed patients who had received 2 to 4 previous lines of therapy. Patients who did not respond to standard-of-care treatment were allowed to cross over to the ide-cel arm. With a median follow-up of 30 months, median PFS was 14 months with ide-cel *vs.* 4 months for standard of care (HR 0.49, 95%CI: 0.38-0.63). Adjusting for the crossover, OS was also improved in patients who received ide-cel (HR 0.72, 95%CI: 0.49-1.01)^[11,83]^.

Even more promising are the results for the second approved CAR-T cell product, cilta-cel. Cilta-cel was approved in February 2022 by the FDA and in May 2022 by the EMA based on the data of the phase 1/2 Cartitude-1 trial (NCT03548207). Patients enrolled in the Cartitude-1 trial had received a median of 6 prior lines of therapy and 88% were triple-class refractory. With a follow-up of more than 27 months, median PFS and OS were not reached. PFS rates were 55% at 27 months. At the same time point, OS rates were 70%^[4,82]^. Similarly to what was seen for ide-cel, the promising data of phase 1/2 were confirmed in the phase 3 Cartitude-4 trial (NCT04181827). Cartitude-4 enrolled lenalidomide refractory patients who had received 1 to 3 prior lines of therapy. Patients were randomized between cilta-cel and standard of care. Median PFS was not reached in the cilta-cel group and was 12 months in the standard of care group (HR 0.26, 95%CI: 0.18-0.38)^[12]^. Recently, updated data were presented to the FDA during the oncologic drugs advisory committee held on March 15th, 2024. During the audition, an OS of 79% at 2 years was reported, with an HR of 0.57 *vs.* standard of care (https://www.fda.gov/media/176988/download).

Despite these encouraging results, PFS curves still do not show a plateau, indicating that most patients will relapse even after TCR therapy.

### TCR therapy mechanism of resistance

Resistance to CAR-T cell therapy and bispecific antibodies can develop through various mechanisms, involving alterations both in the MM cells and in the tumor microenvironment.

Antigen escape, characterized by downregulation or complete loss of the expression of the target antigens, enables MM cells to evade recognition and elimination by CAR-T cells or bispecific antibodies.

Resistance due to antigen loss does not affect the different compounds in equal measure, and it is highly dependent on the target antigen. Despite having been reported, loss of BCMA expression is uncommon at the time of progression^[5,84,85]^. BCMA is encoded by the *TNFRSF17* gene, which is found on chromosome 16p. In 2021, two groups independently reported biallelic BCMA loss as a mechanism driving CAR-T cell resistance^[86,87]^. Using single-cell genomics, Samur *et al.* identified a clone with a biallelic loss of BCMA acquired by deletion of one allele and a mutation that created an early stop codon on the second allele in one patient relapsing 9 months after BCMA CAR-T cell therapy^[86]^. The Würzburg group, on the other hand, reported a patient with homozygous deletion of BCMA at the time of progression after BCMA CAR-T cell therapy^[87]^. Interestingly, the authors also found heterozygous BCMA loss or monosomy of chromosome 16 in 28 of 33 patients who had not been treated with BCMA-targeted therapy^[87]^. This finding has obvious repercussions for clinical practice, as these patients might be more likely to develop homozygous antigen loss following BCMA-targeted therapy. Recently, a different mechanism of BCMA antigen escape was reported, namely a functional epitope loss. Functional epitope loss occurs when mutations or in-frame deletions in the extracellular domain of BCMA occur. These mutations, being non-truncating, do not change the surface expression of BCMA, but affect the binding affinity and, therefore, the efficacy of anti-BCMA-targeted therapies^[27]^. Interestingly, not all TCR therapies are affected in the same way by these extracellular domain mutations, with some compounds still retaining binding capacity and efficacy. For example, Lee *et al.* showed that the presence of the mutation R27P in the extracellular domain of BCMA conferred *in vitro* resistance to the BCMA bispecific antibodies teclistamab and elranatamab, but not to the bispecific antibody alnuctamab or CAR-T cells^[27]^. This suggests that not only the presence of a BCMA mutation, but also the type of mutation will become relevant when assessing patients at relapse for further therapies.

Differently from BCMA, reduction or loss of GPRC5D expression seems to be a common mechanism of resistance. GPRC5D can be lost due to biallelic deletions or single copy number loss^[27]^. GPRC5D loss was reported in all six cases progressing after anti-GPRC5D CAR-T therapy^[88]^. Four additional patients relapsing after anti-GPRC5D bispecific antibodies had GPRC5D biallelic loss at the time of relapse. In two of these cases, the loss of GPRC5D was due to convergent evolution with different subclones losing GPRC5D through mutually exclusive events^[27]^, confirming the pivotal role of intra-clonal heterogeneity in the development of treatment resistance^[89]^. Interestingly, modulation of cereblon with CELMoDs has been suggested to prevent relapse driven by GPRC5D-negative MM cells^[90]^.

Trogocytosis, i.e., the transfer of the target antigen from the tumor cell surface to the CAR-T cells, may also contribute to resistance, on one side, reducing antigen expression on the target cell, and on the other hand, leading to CAR T cell fratricide and thus reducing the activity of CAR-T cells^[91]^.

Tumor load has also been suggested to impact response to TCR therapy, both of CAR-T cells and bispecific antibodies. A high tumor load, leading to chronic antigen exposure, may result in T cell exhaustion, impairing antitumor activity^[92-94]^.

Continuous exposure to the antigen, induced by therapy with bispecific antibodies, also induces T cell exhaustion^[95]^, reducing the efficacy of bispecific antibodies, but also the efficacy of CAR-T cell therapy if T cells are collected immediately after therapy with bispecific antibodies. Clinical trials confirmed that response to BCMA CAR-T cells after BCMA-targeted therapy is reduced, compared to BCMA naïve patients. Patients with the worst response and survival were those who had been treated with bispecific antibodies before CAR-T cell therapy^[96]^. This is likely due to the apheresis of exhausted T cells, which results in less active CAR-T cells after transfection.

An increase in soluble BCMA might also play a role in the development of resistance, specifically resistance to bispecific antibodies^[97,98]^. The mechanisms of these increased resistances are still not completely understood. Possible mechanisms can be the trapping of bispecific antibodies in serum, due to their binding to soluble BCMA, but also an enhancement of gamma-secretase activity, resulting in downregulation of surface BCMA^[97,98]^.

Important in the development of resistance to TCR therapy is the immunosuppressive tumor microenvironment. An immunosuppressive microenvironment can hinder the activity of CAR-T cells and bispecific antibodies. Factors such as an increased expression of inhibitory checkpoint molecules (e.g., PD-L1), the presence of immunosuppressive cells (e.g., regulatory T cells, myeloid-derived suppressor cells), and the secretion of immunosuppressive cytokines (e.g., TGF-β, IL-10) can impair the function of CAR-T cells and diminish the efficacy of bispecific antibodies^[99,100]^. A correlative study of the MonumenTAL1 trial identified lower T cell counts, higher frequency of Treg, and higher expression of inhibitory markers such as lymphocyte activating 3 (LAG-3) and TIM-3 on CD8+ T cells of patients not responding to talquetamab^[101]^. Recently, Friedrich *et al.* showed that the preexisting T cell landscape is pivotal in determining the response to bispecific antibodies. Patients not responding to bispecific antibodies had a higher proportion of exhausted-like CD8+ clones before the start of therapy compared to responding patients. They could also show that bispecific antibodies can also lead to the differentiation and priming of naïve T cells via MHC class I, increasing the number of T cells that can effectively kill MM cells. According to their hypothesis, the presence of MHC class I increases cell recognition and T cell stimulation besides the activation provided by the engagement of the target antigen. The loss of MHC class I can, therefore, be an additional mechanism of resistance to bispecific antibodies, mediating immune escape beyond antigen loss^[102]^.

**Figure 2:**
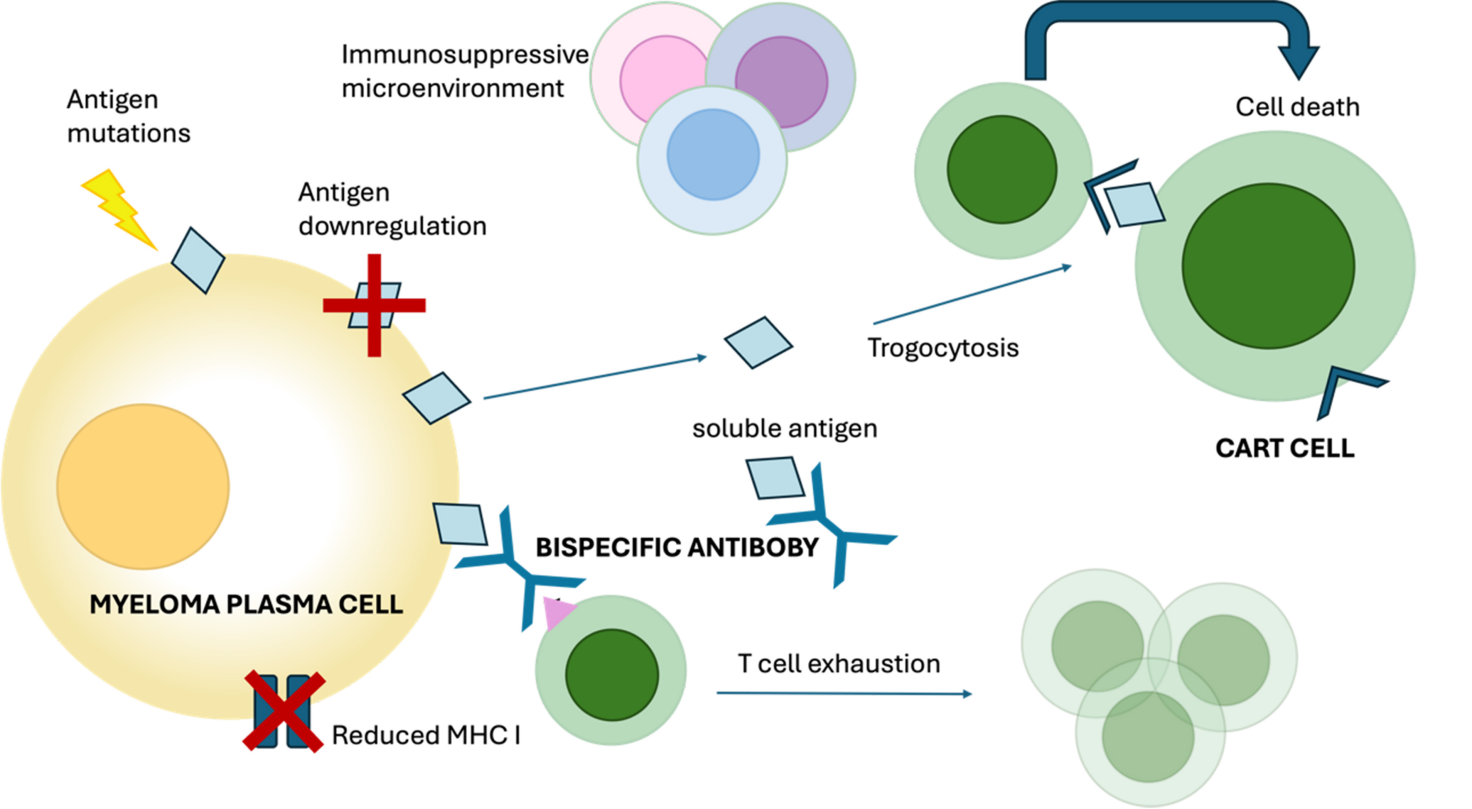
Schematic representation of the main mechanisms of resistance to TCR therapy. Mutation or downregulation of the target antigen led to resistance due to failed recognition of the myeloma cells from the T cells. High tumor burden and high levels of soluble antigen can reduce the efficacy of bispecific antibodies by trapping them before they can reach the target cell. Shedding of the antigen from the target cell to the effectory cell can cause trogocytosis, leading to fratricide of the T cells. Immune exhaustion and an immunosuppressive microenvironment also contribute to disease resistance. For detailed explanations of T cell redirecting therapy mechanisms of resistance, see the main text. TCR: T cell redirecting; MHC I: Major histocompatibility complex class I.

Figure 2 illustrates the main mechanisms of resistance to bispecific antibodies and CAR-T cells.

## CEREBLON MODULATING AGENTS (CELMODS)

CELMoDs are a new class of agents that work by binding to the regulatory protein cereblon (CRBN). CRBN is a component of the Cul4A/DDB1/Roc1 (Cul4A^CRBN^) E3 ubiquitin ligase. E3 ubiquitin ligase is responsible for polyubiquitination and subsequent degradation of substrate proteins. CELMoDs, by binding to CRBN, modulate the function of the E3 ligase complex and can trigger the ubiquitination and subsequent degradation of proteins important for MM cell survival, such as Ikaros and Aiolos^[103]^. Chemically, CELMoDs and IMiDs share similar structures, with glutarimide rings that bind to the CRBN pocket and isoindolinone rings that are responsible for the interaction of CRBN with its substrates. Despite these similarities, CELMoDs structures are more complex than those of IMiDs: to enhance the interaction with CRBN or substrates, CELMoDs also contain additional phenyl and morpholino moieties^[104,105]^. This more complex structure leads to a higher potency of CELMoDs compared to IMiDs, with a 10-20-fold higher affinity to CRBN and more effective degradation of Ikaros and Aiolos^[106,107]^. Additionally, CELMoDs, as well as IMiDs, can stimulate the patient immune system, activating it against myeloma cells. The CELMoDs iberdomide induces depletion of B cells, increases interleukin-2 and interferon-γ production, and can stimulate the activity of T cells and the proliferation of natural killer (NK) cells^[108,109]^. Treatment with iberdomide enhances innate and adaptive immune responses by increasing effector T and NK cells^[110]^. This stimulation of the immune system makes CELMoDs attractive compounds for combination therapy with other immunomodulatory compounds such as monoclonal antibodies or T cell engagers. Preclinical data showed that iberdomide can enhance daratumumab-mediated cytotoxicity via upregulation of both complement-dependent cytotoxicity and antibody-dependent cellular cytotoxicity^[111]^.

CELMoDs are still not approved for routine clinical use, however, due to the above-described intriguing mechanism of action and possible synergies, a wide range of studies investigating these compounds in different settings are ongoing. For example, the phase 1/2 study CC-220-MM-001 (NCT02773030) investigates different combinations of iberdomide. The results from the cohort of iberdomide and dexamethasone have recently been published^[7]^. This cohort enrolled heavily pretreated RRMM patients, including a high proportion of triple refractory patients (refractory to an IMiD, a PI, and an anti CD38 monoclonal antibody) after at least three lines of therapy. Iberdomide showed an ORR of 32%, with 10% of the patients achieving at least a very good partial response (VGPR). PFS and OS were 3 and 11 months, respectively. Importantly, the rate of grade 3 or higher non-hematological side effects was relatively low, with the major toxicity of iberdomide being hematological^[7]^.

Treatment with the CELMoDs iberdomide seems to be able to rescue, at least in part, resistance to other compounds. The combination of iberdomide, daratumumab, and dexamethasone showed an ORR of 35% in 19 patients, of whom 63% were daratumumab-refractory and 58% quad-class refractory^[112]^.

The favorable safety profile of iberdomide prompted its investigation in the maintenance setting. The EMN26 trial (NCT04564703) is a phase 2 study evaluating different doses of iberdomide (0.75, 1.0, 1.3 mg) in the maintenance setting. Early data on the first 69 evaluable patients showed a deepening of the responses after 6 cycles of maintenance therapy. Improvement of response was seen in more than 40% of patients and the PFS at 6 months was above 90%^[113]^. As seen in the previous paragraph, resistance to TCR therapy, including resistance to BCMA-targeted therapy does occur. Iberdomide has also shown its effectiveness in patients previously exposed to BCMA-targeted therapy. A subanalysis of the CC-220-MM-001 trial evaluated 38 patients who received iberdomide after having been exposed to BCMA-targeted therapy, showing an ORR of 37% (including 29% at least a VGPR) and a median PFS of 2.4 months^[114]^.

The second CELMoDs being currently investigated in the clinic is mezigdomide. The CC-92480-MM-001 (NCT03374085) trial is an ongoing phase 1/2 trial evaluating mezigdomide alone or in combination with dexamethasone in triple-class refractory myeloma patients that have received at least three prior lines of therapy. Preliminary data of the first 101 patients reported an ORR of 40%, with 23% of patients achieving at least a VGPR. The median PFS was 4.6 months. The presence of plasmocytomas or pretreatment with BCMA-targeted therapy did not seem to affect the response rate^[115]^. The CC-92480-MM-002 (NCT03989414) evaluates mezigdomide with different treatment combinations in RRMM. In patients with 2 to 4 prior lines of therapy, the combination of mezigdomide with daratumumab and dexamethasone showed an ORR of 75%, with 37% of at least a VGPR. In the combination with elotuzumab, ORR was 36% and 56% for patients receiving 0.3 mg and 0.6 mg of mezidgomide, respectively^[116]^.

### CELMoDs mechanism of resistance

As they share the same target, resistance mechanisms to CELMoDs are similar to those reported for IMiDs. Resistance mechanisms of IMiDs have been widely investigated, and reviewed in^[117]^. Here, we focus on the mechanisms that are more specific to resistance to the newer CELMoDs, while also touching upon resistance mechanisms related to IMiDs. One of the major mechanisms of IMiDs/CELMoD resistance is alterations in the CRBN pathways, such as decreased CRNB expression or mutations in the *CRBN* gene. A recent CRISPR-Cas9 resistance screen against 170 relapse-specific mutations showed that mutations functionally linked to lenalidomide resistance are restricted to those linked to the cereblon E3 ligase complex^[118]^. The importance of genetic alterations of CRBN in the development of resistance to IMiDs/CELMoDs is confirmed by the fact that the rate of alterations increases to up to 30% in relapse patients, while these are very rarely found in newly diagnosed patients^[119,120]^. Interestingly, not all mutations or genetic alterations seem to have the same effect for the different IMiDs/CELMoDs. A recent functional investigation of 12 missense mutations occurring in CRBN showed that mutations in the tri-tryptophan binding pocket or close to the neo-substrate binding area completely abrogated the effects of IMiDs and CELMoDs. On the other hand, mutations in the Lon protease-like domain did not seem to affect IMiDs and CELMoDs sensitivity. Interestingly, some of the mutations conferring resistance to lenalidomide and pomalidomide were still sensitive (at least in part) to iberdomide and mezigdomide, likely due to the different chemical structures of CELMoDs. The authors postulated that, due to the different structures, CELMoDs might be able to overcome some CRBN structural changes conferred by specific mutations^[121]^. A small analysis of five patients treated with mezigdomide in the CC-92480-MM-001 trial (NCT03374085) identified 3p26 loss (encoding for the *CRBN* gene) in both patients who relapsed and those who were primary progressive. While patients who responded and later relapsed had a monoallelic loss of 3p26, the two primary refractory patients had a biallelic loss of 3p26 or a monoallelic loss with the presence of a R309H mutation in the CRBN DDB1 binding domain. Subsequent experiments *in vitro* confirmed that the presence of R309H was able to confer resistance to mezigdomide^[122]^.

Additional mechanisms of decreased CRBN expression/function can be related to epigenetic regulation^[123]^, and combining epigenetic drugs with IMiDs/CELMoDs can be a promising strategy to overcome resistance^[124]^.

A recent mass spectrometry analysis of IMiDs- and CELMoDs-resistant cell lines showed common changes in protein components of the lipid synthesis pathway and identified SCD and MBTPS1 as potential vulnerabilities in iberdomide-resistant cell lines^[125]^. Inhibition of MBTPS1 increased iberdomide sensitivity in iberdomide-resistant NCI-H929 cells, although this effect could not be replicated in the iberdomide-resistant MM1.S cell line^[125]^, indicating that mechanisms of resistance to CELMoDs might vary across different cells.

Additional mechanisms involved in resistance to IMiDs/CELMoDs include alteration of CRBN pathway proteins such as Ikaros, Aiolos, IRF4, and Cullin 4B (CUL4B). In fact, mutations in these genes have been found in circa 10% of IMiD-resistant patients^[126]^. The immune system also plays a role in resistance to IMiDs and CELMoD. Relapse during lenalidomide maintenance can be associated with an increase in immune exhaustion markers after transplantation^[127,128]^. Deep immune profiling on IMiD-resistant patients has shown an expansion of exhausted effector T cell populations, and LAG-3, a marker of T cell exhaustion, has been associated with a reduced PFS^[129,130]^. Although the more potent CELMoDs seem to partially overcome the negative effect of an exhausted immune microenvironment, a defective or exhausted immune system might contribute to the development of resistance to CELMoDs, similar to what is seen in IMiDs^[130]^.

**Figure 3:**
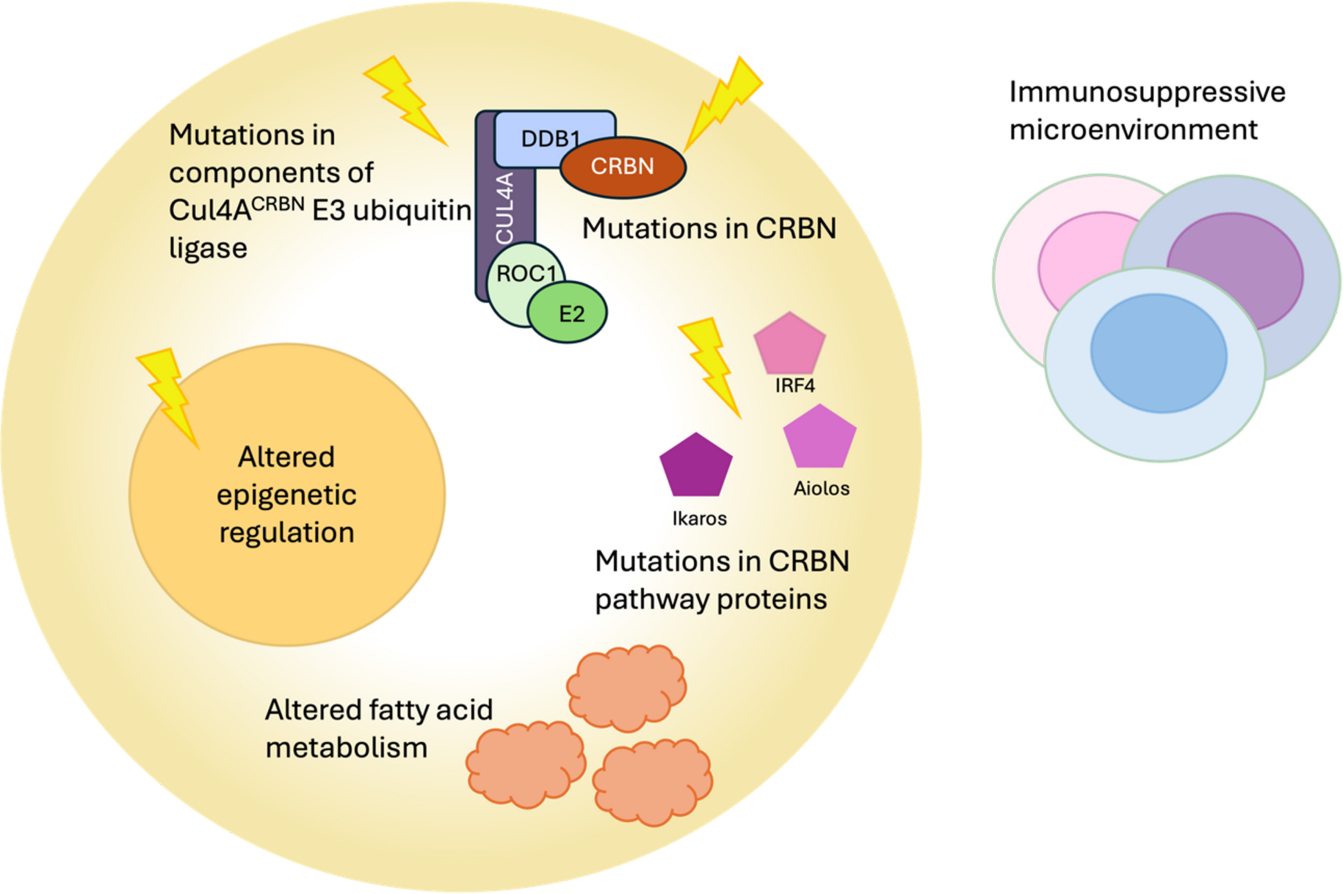
Schematic representation of the main mechanism of resistance to CELMoD therapy. Mutations of cereblon or one of the other components of the CUL4A^CRBN^ E3 ubiquitin ligase are the main mechanisms of resistance to CELMoDs. Additionally, mutations and alterations in downstream proteins of the CRBN pathway also reduce the efficacy of CELMoDs. Altered fatty acid metabolism and epigenetic regulation increase resistance to CELMoDs. An immunosuppressive microenvironment has been linked to reduced PFS in patients receiving IMiDs and CELMoDs. For detailed explanations of mechanisms of resistance to CELMoDs, see the main text. CELMoD: Cereblon E3 ligase modulatory drug; CUL4A: Cullin 4; CRBN: cereblon; PFS: progression-free survival; IMiDs: immunomodulatory drugs; DDB1: DNA damage-binding protein 1, ROC1: ring box 1; IRF4: interferon regulatory factor 4.

Figure 3 illustrates the main mechanism of resistance to CELMoDs.

## COMBINATION THERAPY AND STRATEGIES TO OVERCOME DISEASE RESISTANCE

To overcome or prevent disease resistance, different combination strategies have been used or are currently being assessed within clinical trials. As stated in the section above, the majority of current evidence is focused on how SINE could prevent the development of treatment resistance or on how treatment with SINE could restore sensitivity to other drugs^[29,52-55]^. Recently, a phase II trial showed that treatment with selinexor in combination with carfilzomib, daratumumab, or pomalidomide can partially restore sensitivity to these drugs. In the 18 evaluable patients (of the 20 enrolled), ORR was 33% with a PFS of 5.98 months. These data are very promising, considering that the trial enrolled patients who had progressed under carfilzomib, pomalidomide, or daratumumab-containing regimen, and that the only change was the switch of the companion drug to selinexor^[131]^. On the other hand, combination therapies could reduce the development of resistance to SINE. The already cited phase 1/2 STOMP (NCT02343042) evaluates 12 different combinations of selinexor with standard-of-care backbones. Besides the already reported data on the combinations of selinexor and carfilzomib and selinexor and bortezomib (see paragraph SINE), data on the combinations of selinexor with daratumumab and selinexor with pomalidomide have also been reported [Table 1]. The combination of selinexor with daratumumab (XDd) had an ORR of 69%, with a median PFS of 12.5 months. These results are very interesting, considering that all the patients enrolled had been previously exposed to PIs and IMiDs and 74% were PI- and IMiD-refractory. Only two patients had been previously exposed to daratumumab and were refractory. Both these patients did not respond to the XDd combination^[132]^.

**Table 1:** Ongoing clinical trials of combination strategies with SINE, TCR therapy, and CELMoDs to overcome treatment resistance^*^

**Trial**	**NCT**	**Phase**	**Type of patients**	***N*. patients**	**Doses (if different from standard approved dose)**	**ORR**	**PFS**	**Major toxicities grade 3**	**Ref.**
Selinexor									
XKd	NCT02343042	Ib/II	RRMM	32	100 mg QW	78.1%	15 mo	Neutropenia 6%, thrombocytopenia 25%, anemia 19%, nausea (6%), fatigue (9%)	[56]
XVd	NCT02343042	Ib/II	RRMM	42	80-100 mg QW / 60-80 BIW	63%	9 mo	Neutropenia 24%, anemia 12%, thrombocytopenia 45%, diarrhea 7%, fatigue 14%	[57]
XDd	NCT02343042	I/IIb	RRMM	34	100 mg QW / 60 mg BIW	69%	12.5 mo	Neutropenia 27%, thrombocytopenia 47%, anemia 32%, nausea 9%, fatigue 18%	[132]
XPd	NCT02343042	Ib/II	RRMM	20/19	60 mg QW / 40 mg QW	65%/42%	10 mo / 18 mo	Neutropenia 60%/58%, thrombocytopenia 25%/11%, anemia 25%/5%, nausea all grades 70%/26%, fatigue 15%/5%	[133,134]
XVd *vs.* Vd	NCT03110562	III	RRMM	195	100 mg dose on days 1, 8, 15, 22, and 29 of each 5-week cycle	76%	NA	Neutropenia 9%, thrombocytopenia 39%, anemia 16%, fatigue 13%, nausea 8%, diarrhoea 6%	[58]
XKd, XPd, XDd	NCT04661137	II	RRMM refractory to K, Pom or D	20	80 mg in K arm, 60 mg in Pom arm, 100 mg in D arm	33%	6 mo	Neutropenia 25%, thrombocytopenia 15%, pneumonia 10%	[131]
XPd *vs.* EloPd (EMN29)	NCT05028348	III	RRMM	Planned 222	40 mg QW	Trial ongoing	Trial ongoing	Trial ongoing	Clinicaltrial.gov, accessed 27 May 2024
Teclistamab									
TecDR (MajesTEC2)	NCT04722146	I	RRMM	32	0.72 mg/kg / 1.5 mg/kg	100%/81%	NA	Neutropenia 69%, anemia 43%, infections 29%, CRS 0%, ICANS 0%	[144]
TecTal (± Dara) (RedirecTT-1)	NCT04586426	Ib/II	RRMM	63 (Planned 208)		84% (73% in EMM)	NA	Neutropenia 75%, anemia 43%, infections 53%, CRS 3%, ICANS 1pt	[145,146]
TecDara (TRIMM-2)	NCT04108195	I	RRMM	37		78%	NA	Neutropenia 50%, thrombocytopienia 28%, anemia 28%, infections 28%, CRS 0%, ICANS 0%	[154]
MajesTEC2 other cohorts (TecPomDara; TecDVR; TecLen; Tec + Nirogacestat)	NCT04722146	I	RRMM	Planned 140	Trial ongoing	Trial ongoing	Trial ongoing	Trial ongoing	Clinicaltrial.gov, accessed 27 May 2024
TRIMM-2 other cohort TecPomDara	NCT04108195	Ib	RRMM	Planned 289	Trial ongoing	Trial ongoing	Trial ongoing	Trial ongoing	Clinicaltrial.gov, accessed 27 May 2024
Limited duration teclistamab	NCT05932680	II	RRMM	Planned 75	Teclistamab discontinuation after 6 to 9 months for pts > VGPR	Trial ongoing	Trial ongoing	Trial ongoing	Clinicaltrial.gov, accessed 27 May 2024
Tec + PD-1 inhibitor (TRIMM-3)	NCT05338775	I	RRMM	Planned 152	Trial ongoing	Trial ongoing	Trial ongoing	Trial ongoing	Clinicaltrial.gov, accessed 27 May 2024
TecDara *vs.* DPd or DVd (MajesTEC3)	NCT05083169	III	RRMM	Planned 587	Trial ongoing	Trial ongoing	Trial ongoing	Trial ongoing	Clinicaltrial.gov, accessed 27 May 2024
TecDRd *vs.* TecDVRd (Majestec5, GMMG-HD10/DSMM-XX)	NCT05695508	II	NDMM	Planned 70	Trial ongoing	Trial ongoing	Trial ongoing	Trial ongoing	Clinicaltrial.gov, accessed 27 May 2024
TecDR *vs.* DRd (MajesTEC7)	NCT05552222	III	Elderly NDMM	Planned 1590	Trial ongoing	Trial ongoing	Trial ongoing	Trial ongoing	Clinicaltrial.gov, accessed 27 May 2024
TecLen or TecDara (IFM2021-01)	NCT05572229	II	Elderly NDMM	Planned 74	Trial ongoing	Trial ongoing	Trial ongoing	Trial ongoing	Clinicaltrial.gov, accessed 27 May 2024
Talquetamab									
TalPom (MonumenTAL2)	NCT05050097	I	RRMM	35	Pom 2 mg from cycle 2	87%	6-months PFS 93%	Neutropenia 49%, thrombocytopenia 20%, anemia 26%, CRS 3%, infections 23%, dysgeusia 77% (all grades), nail and skin 66% (all grades)	[155]
TalDara (TRIMM-2)	NCT04108195	I	RRMM	65		78%	19.4 mo	Neutropenia 26%, CRS 0%, infections 25%, dysgeusia 75% (all grades), nail and skin 55% (all grades)	[156]
MonumenTAL2 other cohorts (TalK, TalKD, TalLen, TalDR)	NCT05050097	I	RRMM	Planned 182	Trial ongoing	Trial ongoing	Trial ongoing	Trial ongoing	Clinicaltrial.gov, accessed 27 May 2024
TalDara ± Pom vd DPd (MonumenTAL3)	NCT04108195	III	RRMM	Planned 290	Trial ongoing	Trial ongoing	Trial ongoing	Trial ongoing	Clinicaltrial.gov, accessed 27 May 2024
TalPom or TecTal *vs.* EloPd or VPd (MonumenTAL6)	NCT06208150	III	RRMM	Planned 795	Trial ongoing	Trial ongoing	Trial ongoing	Trial ongoing	Clinicaltrial.gov, accessed 27 May 2024
TRIMM-2 other cohort TalPomDara	NCT04108195	Ib	RRMM	Planned 289	Trial ongoing	Trial ongoing	Trial ongoing	Trial ongoing	Clinicaltrial.gov, accessed 27 May 2024
Tal + PD-1 inhibitor (TRIMM-3)	NCT05338775	I	RRMM	Planned 152	Trial ongoing	Trial ongoing	Trial ongoing	Trial ongoing	Clinicaltrial.gov, accessed 27 May 2024
TalDR *vs.* DRd (MajeTEC7)	NCT05552222	III	Elderly NDMM	Planned 1590	Trial ongoing	Trial ongoing	Trial ongoing	Trial ongoing	Clinicaltrial.gov, accessed 27 May 2024
Elranatamab									
Elra + nirogacestat, ElraRd (MagnetisMM4)	NCT05090566	I/II	RRMM	Planned 105	Trial ongoing	Trial ongoing	Trial ongoing	Trial ongoing	Clinicaltrial.gov, accessed 27 May 2024
Elra *vs.* ElraDara *vs.* DPd (MagnetisMM5)	NCT05020236	III	RRMM	Planned 762	Trial ongoing	Trial ongoing	Trial ongoing	Trial ongoing	Clinicaltrial.gov, accessed 27 May 2024
ElraDaraLen *vs.* DRd (MagnetisMM6)	NCT05623020	III	Elderly NDMM	Planned 966	Trial ongoing	Trial ongoing	Trial ongoing	Trial ongoing	Clinicaltrial.gov, accessed 27 May 2024
ElraKd, Elra + maplirpacept (MagnetisMM20)	NCT05675449	I	RRMM	Planned 14	Trial ongoing	Trial ongoing	Trial ongoing	Trial ongoing	Clinicaltrial.gov, accessed 27 May 2024
Iberdomide									
IberKd	NCT05199311	I/II	Transplant eligible NDMM	13	Iber 1.0, 1.3, 1.6 mg	100%	NA	Neutropenia 8%, maculopapular rash 8%	[157]
IberDd	NCT02773030	I/II	RRMM	43	Iber 1.0, 1.3, 1.6 mg	46%	DOR NR	Neutropenia 67%, thrombocytopenia 13%, anemia 21%, infections 15%	[158]
IberVd	NCT02773030	I/II	RRMM	25	Iber 1.0, 1.3, 1.6 mg	56%	DOR 36 weeks	Neutropenia 28%, thrombocytopenia 24%, anemia 12%, infections 20%	[152]
IberKd	NCT02773030	I/II	RRMM	9	Iber 1.1, 1.3 mg	56%	NA	Neutropenia 33%, thrombocytopenia 11%, infections 33%, fatigue 11%	[152]
IberVd	NCT02773030	I/II	NDMM	18	Iber 1.6 mg	89%	NA	Neutropenia 18%, thrombocytopenia NA, anemia NA, infections 19%, PNP 12%	[159]
IberVd ± Isa	NCT05272826	II	Elderly NDMM	Planned 75	Trial ongoing	Trial ongoing	Trial ongoing	Trial ongoing	Clinicaltrial.gov, accessed 27 May 2024
IberDVd	NCT05392946	I/II	NDMM	Planned 18	Trial ongoing	Trial ongoing	Trial ongoing	Trial ongoing	Clinicaltrial.gov, accessed 27 May 2024
IberDd	NCT05527340	II	Elderly NDMM	Planned 140	Trial ongoing	Trial ongoing	Trial ongoing	Trial ongoing	Clinicaltrial.gov, accessed 27 May 2024
IberCd	NCT04392037	II	RRMM	60	Trial ongoing	Trial ongoing	Trial ongoing	Trial ongoing	Clinicaltrial.gov, accessed 27 May 2024
IberIxad (I2D IFM2021_03)	NCT04998786	II	Elderly first relapse	80	Trial ongoing	Trial ongoing	Trial ongoing	Trial ongoing	Clinicaltrial.gov, accessed 27 May 2024
IberDKd	NCT05896228	II	RRMM	30	Trial ongoing	Trial ongoing	Trial ongoing	Trial ongoing	Clinicaltrial.gov, accessed 27 May 2024
IberElod	NCT05560399	I	RRMM	6	Trial ongoing	Trial ongoing	Trial ongoing	Trial ongoing	Clinicaltrial.gov, accessed 27 May 2024
Iber + elranatamab (MagnetisMM-30)	NCT06215118	I	RRMM	100	Trial ongoing	Trial ongoing	Trial ongoing	Trial ongoing	Clinicaltrial.gov, accessed 27 May 2024
Iber + cevostamab	NCT05583617	I/II	RRMM	200^§^	Trial ongoing	Trial ongoing	Trial ongoing	Trial ongoing	Clinicaltrial.gov, accessed 27 May 2024
Iber + GPRC5D-targeted CAR-T cell therapy BMS-986393	NCT06121843	I	RRMM	11	Trial ongoing	Trial ongoing	Trial ongoing	Trial ongoing	Clinicaltrial.gov, accessed 27 May 2024
Mezigdomide									
MeziVd		I/II	RRMM	77	Mezi 0.3, 0.6, 1.0 mg	75%/84%/91%	DOR 10.9 months / NR / NR	Neutropenia 59%, thrombocytopenia 27%, anemia 6%, infections 33%, PNP 5%	[160]
MeziKd		I/II	RRMM	27	Mezi 0.3, 0.6, 1.0 mg	85%	DOR 12.3 mo	Neutropenia 41%, thrombocytopenia 19%, anemia 15%, infections 30%	[160]
MeziDd		I/II	RRMM	57	Mezi 0.3, 0.6 mg	75%	NA	Neutropenia 54%, thrombocytopenia 7%, anemia 11%, infections 20%	[116]
MeziElod		I/II	RRMM	20	Mezi 0.3, 0.6 mg	45%	NA	NA	[116]
MeziIxad	NCT06050512	I/II	RRMM	Planned 34	Trial ongoing	Trial ongoing	Trial ongoing	Trial ongoing	Clinicaltrial.gov, accessed 27 May 2024
MeziElod	NCT05981209	I	RRMM after anti-CD38 and anti-BCMA therapies	Planned 27	Trial ongoing	Trial ongoing	Trial ongoing	Trial ongoing	Clinicaltrial.gov, accessed 27 May 2024
Mezi post IdeCel	NCT06048250	I	RRMM	Planned 15	Trial ongoing	Trial ongoing	Trial ongoing	Trial ongoing	Clinicaltrial.gov, accessed 27 May 2024
Mezi + alnuctamab	NCT06163898	I/II	RRMM	Planned 156	Trial ongoing	Trial ongoing	Trial ongoing	Trial ongoing	Clinicaltrial.gov, accessed 27 May 2024
Mezi + GPRC5D-targeted CAR-T cell therapy BMS-986393	NCT06121843	I	RRMM	Planned 111	Trial ongoing	Trial ongoing	Trial ongoing	Trial ongoing	Clinicaltrial.gov, accessed 27 May 2024

^*^Maintenance studies have not been included; where the main partner of the therapy is a bispecific antibody, only already approved bispecific antibodies have been included. ^§^Including lenalidomide arm. SINE: Selective inhibitors of nuclear export; TCR: T cell redirecting; CELMoDs: cereblon E3 ligase modulatory drugs; ORR: overall response rate; PFS: progression-free survival; XKd: selinexor, carfilzomid, dexamethasone; RRMM: relapsed and/or refractory multiple myeloma; QW: once a week; mo: months; XVd: selinexor, bortezomib, dexamethasone; BIW: twice a week; XDd: selinexor, daratumumab, dexamethasone; XPd: selinexor, pomalidomide, dexamethasone; Vd: bortezomib, dexamethasone; K: carfilzomib; Pom: pomalidomide; D: daratumumab; EloPd: elotuzumab, pomalidomide, dexamethasone; TecDR: teclistamab, lenalidomide, daratumumab; NA: not assessed; CRS: cytokine release syndrome; ICANS: immune effector cell-associated neurotoxicity syndrome; TecTal: teclistamab, talquetamab; EMM: extramedullary myeloma; TecDara: teclistamab, daratumumab; TecPomDara: teclistamamb, pomalidomide, daratumumab; TecDVR: teclistamab, daratumumab, bortezomib, lenalidomide; TecLen: teclistamab, lenalidomide; Tec: teclistamab; VGPR: very good partial response; DPd: daratumumab, pomalidomide, dexamethasone; DVd: daratumumab, bortezomib, dexamethasone; DRd: daratumumab, lenalidomide, dexamethasone; NDMM: newly diagnosed multiple myeloma; TalPom: talquetamab, pomalidomide; TalK: talquetamab, carfilzomib; TalKD: talquetamab, carfilzomib, daratumumab; TalLen: talquetamad, lenalidomide; TalDR: talquetamad, daratumumab, lenalidomide; TalDara: talquetamab, daratumumab; DPd: daratumumab, pomalidomide, dexamethasone; VPd: bortezomib, pomalidomide, dexamethasone; TalPomDara: talquetamab, pomalidomide, daratumumab; Tal: talquetamab; Elra: elranatamb; ElraRd: elranatamab, lenalidomide, dexamethasone; ElraDaraLen: elranatamab, lenalidomide, dexamethasone; IberKd: iberdomide, carfilzomib, dexamethasone; IberDd: iberdomide, daratumumab, dexamethasone; DOR: duration of response; NR: not reached; IberVd: iberdomide, bortezomib, dexamethasone; Isa: isatuximab; IberDVd: iberdomide, daratumumab, bortezomib, dexamethasone; IberDd: iberdomide, daratumumab, dexamethasonse; IberCd: iberdomide, cyclophosphamide, dexamethasone; IberIxad: iberdomide, ixazomib, dexamethasone; IberDKd: iberdomide, daratumumab, carfilzomib, dexamethasonse; IberElod: iberdomide, elotuzumab, dexamethasone; Iber: iberdomide; MeziVd: mezigdomide, bortezomib, dexamethasone; MeziKd: mezigdomide, carfilzomib, dexamethasone; MeziDd: mezigdomide, daratumumab, dexamethasone; MeziElod: mezigdomide, elotuzumab, dexamethasone; MeziIxad: mezigdomide, ixazomib, dexamethasone.

The combination of selinexor and pomalidomide was tested with two different doses of selinexor, 60 mg weekly and 40 mg weekly. The ORR was 65% (selinexor dose of 60 mg weekly) and 42% (selinexor 40 mg weekly). The median PFS was longer in the 40 mg arm than in the 60 mg arm (18.4 months *vs.* 9.5 months, respectively). Despite the small number of patients, the data show two important considerations: the first one is that ORR remained high in patients previously exposed to anti-CD38 MoAbs (ORR 64%), and all three patients refractory to pomalidomide responded to the XPd combination. These data suggest that the combination of selinexor with pomalidomide can restore sensitivity to IMiDs and overcome resistance to anti-CD38 MoAbs. The second important point made by the study was that the rate of gastrointestinal adverse events of all grades decreased from 70% to 32% when the selinexor dosage was reduced from 60 to 40 mg weekly, with no impact on PFS^[133,134]^.

Currently, the European Myeloma Network is conducting a trial (EMN29, NCT05028348) aimed at comparing the safety and efficacy of XPd (with selinexor 40 mg weekly) *vs.* the combination of elotuzumab, pomalidomide, and dexamethasone. The study is currently recruiting, and results are eagerly awaited. A summary of trials on selinexor combinations can be found in Table 1.

Different strategies have been suggested and are currently being explored to overcome resistance to bispecific antibodies and CAR-T cells. Besides the already-mentioned promising combination strategies with CELMoDs^[90]^, dual CAR-T cells are currently being developed. The possibility of targetting two antigens simultaneously would increase efficacy and reduce development of resistance^[135-137]^. Other strategies to prevent the development of resistance to CAR-T cells are preventing CAR-T cell exhaustion by optimizing CAR-T cell structure, utilizing naive or central memory T cells, or by inhibiting exhaustion-related signals such as BATF, TGF-β, PD-1, and PI3K by using tyrosine kinase inhibitors such as dasatinib^[138-140]^. Interestingly, histone deacetylase inhibitors such as panobinostat seem to downregulate exhaustion-related genes, and could also have a role in reducing or preventing CAR-T cell exhaustion^[141]^. To reduce the impact of immune exhaustion, current investigations are also exploring therapy-free interval or fixed duration therapy for bispecific antibodies or the early collection of T cells for the later production of CAR-T cells^[142,143]^. A trial currently being conducted at the University of Pennsylvania (LimiTec, NCT05932680) is testing the hypothesis that limited-duration teclistamab (stopped after 6 to 9 cycles in patients achieving at least a VGPR) is non-inferior to the standard continuous administration of teclistamab in RRMM patients. The rationale of the trial is that a limited duration of teclistamab will prevent T cell exhaustion, preserving the efficacy of TCR therapy even in patients relapsing after these therapies. Other strategies to prevent T cell exhaustion are to combine bispecific antibodies with PD1 inhibitors, such as in the ongoing TRIMM-3 (NCT05338775) trial. Immunomodulatory drugs such as pomalidomide and lenalidomide, can potentiate T cell activation. Several trials (NCT05572229, NCT04108195, NCT05552222, NCT05695508) are evaluating the combination of bispecific antibodies with IMiDs, although particular attention must be paid to the development of infections. In the MajesTEC2 trial (NCT04722146), evaluating the combination of teclistamab, lenalidomide, and daratumumab in elderly newly diagnosed myeloma patients, despite a very promising ORR (all 13 patients evaluated responded to the treatment), the rate of infections was 75%^[144]^. This is an important point, as the quest to avoid or revert treatment resistance should not be at the price of increased toxicities. An alternative strategy to prevent T cell exhaustion could be the combination of TCR therapy with cytotoxic agents such as cyclophosphamide^[94]^.

Besides T cell exhaustion, antigen loss is another factor that can induce resistance to TCR therapy. If for CAR-T cells, dual CAR-Ts are being developed^[135-137]^, then for bispecific antibodies, a promising strategy is to combine two antibodies. Preliminary results of the RedirecTT-1 trial (NCT04586426), combining teclistamab and talquetamab, showed very promising results, with an ORR of 84% in all patients. Importantly, ORR was 73% in the 26 patients with extramedullary myeloma, suggesting that the combination of two bispecific antibodies is effective in this difficult-to-treat population^[145,146]^. Additional strategies to overcome antigen loss could be the development of trispecific antibodies that target two antigens on the plasma cells. An example could be JNJ-79635322, a trispecific antibody that targets CD3 on T cells and BCMA and GPRC5D on plasma cells. JNJ-79635322 showed preclinical efficacy, and a phase I clinical trial is currently ongoing (NCT05652335)^[147]^. Additional strategies could be the development of antibodies with a higher affinity to full-length BCMA and not sBCMA, increasing the concentration of bispecific antibodies in patients with high baseline sBCMA levels, and enhancing the density of BCMA molecules on myeloma cells by using gamma-secretase inhibitors^[148]^. Major trials evaluating combination therapy for bispecific antibodies are summarized in Table 1.

Different combination approaches combining CELMoDs with MoAbs and PIs are currently in phase II and III clinical trials and are summarized in Table 1. As seen with other classes of drugs, combination therapies, by affecting myeloma cells in different ways, might reduce the chance of the development of resistance. Preclinical data showing increased apoptosis when mezigomide was combined with PIs or daratumumab support these clinical trials^[149-151]^. As discussed in the previous section and in the previous paragraph [see paragraph CEREBLON MODULATING AGENTS (CELMODS)], due to the immunomodulatory properties of CELMoDs, an attractive combination is the one with bispecific antibodies. Indeed, *in vitro* and *in vivo*, the combination of iberdomide and mezigdomide with the anti-BCMA bispecific antibody alnuctamab or with the anti-GPRC5D forimtamig enhanced antitumor activity and tumor regression^[90,152]^. Iberdomide has also shown an enhancement in CAR-T cell activation and cytokine production, making CELMoDs an interesting maintenance therapy post CAR-T cell treatment to prevent the emergence of treatment resistance^[153]^. Trials on the combination of CELMoDs with PI and MoAb are ongoing, and early results are already available [Table 1]. Additionally, trials testing the combination of CELMoDs and bispecific antibodies and CAR-T cells are currently ongoing, although efficacy and safety data are still pending. Ongoing and planned trials with CELMoDs combinations are summarized in Table 1.

## CONCLUSION

Novel therapies are changing the prognosis of MM. SINE, TCR therapies, and CELMoDs have shown remarkable efficacy; however, the emergence of resistance poses a significant challenge.

Mechanisms of resistance to SINE, TCR therapies, and CELMoDs are intricate and complex, involving both intrinsic and extrinsic mechanisms. From genetic alterations to dysregulated signaling pathways, to the development of an immunosuppressive microenvironment, multiple factors contribute to the development of resistance. A deeper understanding of these mechanisms is pivotal in the quest toward myeloma cure.

Exploring novel therapeutic approaches such as combination therapies and targeted interventions against specific resistance mechanisms is of primary importance to overcome treatment-emergent disease refractoriness. Efforts in this respect are already ongoing, and trials that combine CELMoDs with TCR therapies seem particularly promising. Additionally, the development of predictive biomarkers, such as gene signatures or immune profiling, holds promise in overcoming resistance and improving patient outcomes. Advancements in technology, such as high-throughput screening and computational modeling, can provide invaluable tools for identifying new targets and optimizing treatment regimens. Besides being highly effective, these novel drugs do show adverse events that are somehow different from those reported with other therapies. Examples are the gastrointestinal toxicity of SINE, the neurologic side effects of CAR-T cells, and the high risk of infection seen with bispecific antibodies. As these therapies move to the earlier lines of therapies, these side effects will have to be balanced against the efficacy and the risk of resistance development. Selecting the patients that will profit the most from each therapy, for example evaluating patients’ specific immune profile or the tumor dependency on p53 and protein trafficking, will be pivotal in paving the road toward a truly personalized medicine, where each patient or group of patients will receive a drug combination more suitable for their characteristics.

## DECLARATIONS

### Copyright

© The Author(s) 2024.

## References

[1] Gatopoulou X, Bardenheuer K, Van Hoorenbeeck S, Kempel A (2016). Treatment patterns of relapsed and refractory multiple myeloma in Europe (EU-28). Value Health.

[2] Chari A, Minnema MC, Berdeja JG (2022). Talquetamab, a T-cell-redirecting GPRC5D bispecific antibody for multiple myeloma. N Engl J Med.

[3] Moreau P, Garfall AL, van de Donk NWCJ (2022). Teclistamab in relapsed or refractory multiple myeloma. N Engl J Med.

[4] Berdeja JG, Madduri D, Usmani SZ (2021). Ciltacabtagene autoleucel, a B-cell maturation antigen-directed chimeric antigen receptor T-cell therapy in patients with relapsed or refractory multiple myeloma (CARTITUDE-1): a phase 1b/2 open-label study. Lancet.

[5] Munshi NC, Anderson LD Jr, Shah N (2021). Idecabtagene vicleucel in relapsed and refractory multiple myeloma. N Engl J Med.

[6] Chari A, Vogl DT, Gavriatopoulou M

[7] Lonial S, Popat R, Hulin C (2022). Iberdomide plus dexamethasone in heavily pretreated late-line relapsed or refractory multiple myeloma (CC-220-MM-001): a multicentre, multicohort, open-label, phase 1/2 trial. Lancet Haematol.

[8] Lesokhin AM, Tomasson MH, Arnulf B (2023). Elranatamab in relapsed or refractory multiple myeloma: phase 2 MagnetisMM-3 trial results. Nat Med.

[9] Gandhi UH, Cornell RF, Lakshman A (2019). Outcomes of patients with multiple myeloma refractory to CD38-targeted monoclonal antibody therapy. Leukemia.

[10] Mateos MV, Weisel K, De Stefano V (2022). LocoMMotion: a prospective, non-interventional, multinational study of real-life current standards of care in patients with relapsed and/or refractory multiple myeloma. Leukemia.

[11] Rodriguez-Otero P, Ailawadhi S, Arnulf B (2023). Ide-cel or standard regimens in relapsed and refractory multiple myeloma. N Engl J Med.

[12] San-Miguel J, Dhakal B, Yong K (2023). Cilta-cel or standard care in lenalidomide-refractory multiple myeloma. N Engl J Med.

[13] Solimando AG, Malerba E, Leone P (2022). Drug resistance in multiple myeloma: soldiers and weapons in the bone marrow niche. Front Oncol.

[14] Abe M (2011). Targeting the interplay between myeloma cells and the bone marrow microenvironment in myeloma. Int J Hematol.

[15] Ferrucci A, Moschetta M, Frassanito MA (2014). A HGF/cMET autocrine loop is operative in multiple myeloma bone marrow endothelial cells and may represent a novel therapeutic target. Clin Cancer Res.

[16] Gnoni A, Brunetti O, Longo V (2020). Immune system and bone microenvironment: rationale for targeted cancer therapies. Oncotarget.

[17] Solimando AG, Vacca A, Ribatti D (2020). A comprehensive biological and clinical perspective can drive a patient-tailored approach to multiple myeloma: bridging the gaps between the plasma cell and the neoplastic niche. J Oncol.

[18] Harmer D, Falank C, Reagan MR (2018). Interleukin-6 interweaves the bone marrow microenvironment, bone loss, and multiple myeloma. Front Endocrinol.

[19] Vacca A, Ria R, Ribatti D (2003). A paracrine loop in the vascular endothelial growth factor pathway triggers tumor angiogenesis and growth in multiple myeloma. Haematologica.

[20] Papa S, Choy PM, Bubici C (2019). The ERK and JNK pathways in the regulation of metabolic reprogramming. Oncogene.

[21] Ikeda S, Abe F, Matsuda Y, Kitadate A, Takahashi N, Tagawa H (2020). Hypoxia-inducible hexokinase-2 enhances anti-apoptotic function via activating autophagy in multiple myeloma. Cancer Sci.

[22] Maiso P, Huynh D, Moschetta M (2015). Metabolic signature identifies novel targets for drug resistance in multiple myeloma. Cancer Res.

[23] Bailur JK, McCachren SS, Doxie DB (2019). Early alterations in stem-like/resident T cells, innate and myeloid cells in the bone marrow in preneoplastic gammopathy. JCI Insight.

[24] Giallongo C, Tibullo D, Parrinello NL (2016). Granulocyte-like myeloid derived suppressor cells (G-MDSC) are increased in multiple myeloma and are driven by dysfunctional mesenchymal stem cells (MSC). Oncotarget.

[25] Gabrilovich DI, Nagaraj S (2009). Myeloid-derived suppressor cells as regulators of the immune system. Nat Rev Immunol.

[26] Gavalas NG, Tsiatas M, Tsitsilonis O (2012). VEGF directly suppresses activation of T cells from ascites secondary to ovarian cancer via VEGF receptor type 2. Br J Cancer.

[27] Lee H, Ahn S, Maity R (2023). Mechanisms of antigen escape from BCMA- or GPRC5D-targeted immunotherapies in multiple myeloma. Nat Med.

[28] Turner JG, Dawson J, Sullivan DM (2012). Nuclear export of proteins and drug resistance in cancer. Biochem Pharmacol.

[29] Theodoropoulos N, Lancman G, Chari A (2020). Targeting nuclear export proteins in multiple myeloma therapy. Target Oncol.

[30] Arnaoutov A, Azuma Y, Ribbeck K (2005). Crm1 is a mitotic effector of Ran-GTP in somatic cells. Nat Cell Biol.

[31] Vogt PK, Jiang H, Aoki M (2005). Triple layer control: phosphorylation, acetylation and ubiquitination of FOXO proteins. Cell Cycle.

[32] Pichler A, Melchior F (2002). Ubiquitin-related modifier SUMO1 and nucleocytoplasmic transport. Traffic.

[33] Kojima K, Kornblau SM, Ruvolo V (2013). Prognostic impact and targeting of CRM1 in acute myeloid leukemia. Blood.

[34] Zhou F, Qiu W, Yao R (2013). CRM1 is a novel independent prognostic factor for the poor prognosis of gastric carcinomas. Med Oncol.

[35] Kandarpa M, Kraftson SJ, Maxwell SP (2011). CRM1 is highly expressed in myeloma plasma cells and its inhibition by KPT-SINE induces cytotoxicity by increasing p53 in the nucleus of multiple myeloma (MM) cells. Blood.

[36] Camus V, Miloudi H, Taly A, Sola B, Jardin F (2017). XPO1 in B cell hematological malignancies: from recurrent somatic mutations to targeted therapy. J Hematol Oncol.

[37] Fung HYJ, Niesman A, Chook YM (2021). An update to the CRM1 cargo/NES database NESdb. Mol Biol Cell.

[38] Huang ZL, Gao M, Li QY (2013). Induction of apoptosis by directing oncogenic Bcr-Abl into the nucleus. Oncotarget.

[39] Culjkovic-Kraljacic B, Baguet A, Volpon L, Amri A, Borden KL (2012). The oncogene eIF4E reprograms the nuclear pore complex to promote mRNA export and oncogenic transformation. Cell Rep.

[40] Nikolaev AY, Li M, Puskas N, Qin J, Gu W (2003). Parc: a cytoplasmic anchor for p53. Cell.

[41] Zhang Y, Xiong Y (2001). A p53 amino-terminal nuclear export signal inhibited by DNA damage-induced phosphorylation. Science.

[42] O’Brate A, Giannakakou P (2003). The importance of p53 location: nuclear or cytoplasmic zip code?. Drug Resist Updat.

[43] Rodríguez JA, Henderson BR (2000). Identification of a functional nuclear export sequence in BRCA1. J Biol Chem.

[44] Volpon L, Culjkovic-Kraljacic B, Sohn HS, Blanchet-Cohen A, Osborne MJ, Borden KLB (2017). A biochemical framework for eIF4E-dependent mRNA export and nuclear recycling of the export machinery. RNA.

[45] Culjkovic-Kraljacic B, Fernando TM, Marullo R (2016). Combinatorial targeting of nuclear export and translation of RNA inhibits aggressive B-cell lymphomas. Blood.

[46] Tai YT, Landesman Y, Acharya C (2014). CRM1 inhibition induces tumor cell cytotoxicity and impairs osteoclastogenesis in multiple myeloma: molecular mechanisms and therapeutic implications. Leukemia.

[47] Zheng Y, Gery S, Sun H, Shacham S, Kauffman M, Koeffler HP (2014). KPT-330 inhibitor of XPO1-mediated nuclear export has anti-proliferative activity in hepatocellular carcinoma. Cancer Chemother Pharmacol.

[48] Liu Y, Azizian NG, Dou Y, Pham LV, Li Y (2019). Simultaneous targeting of XPO1 and BCL2 as an effective treatment strategy for double-hit lymphoma. J Hematol Oncol.

[49] Wang JC (2002). Cellular roles of DNA topoisomerases: a molecular perspective. Nat Rev Mol Cell Biol.

[50] Turner JG, Engel R, Derderian JA, Jove R, Sullivan DM (2004). Human topoisomerase IIalpha nuclear export is mediated by two CRM-1-dependent nuclear export signals. J Cell Sci.

[51] Engel R, Valkov NI, Gump JL, Hazlehurst L, Dalton WS, Sullivan DM (2004). The cytoplasmic trafficking of DNA topoisomerase IIalpha correlates with etoposide resistance in human myeloma cells. Exp Cell Res.

[52] Turner JG, Dawson J, Emmons MF (2013). CRM1 inhibition sensitizes drug resistant human myeloma cells to topoisomerase II and proteasome inhibitors both in vitro and ex vivo. J Cancer.

[53] Gandhi UH, Senapedis W, Baloglu E (2018). Clinical implications of targeting XPO1-mediated nuclear export in multiple myeloma. Clin Lymphoma Myeloma Leuk.

[54] Rosebeck S, Alonge MM, Kandarpa M (2016). Synergistic myeloma cell death via novel intracellular activation of caspase-10-dependent apoptosis by carfilzomib and selinexor. Mol Cancer Ther.

[55] Turner JG, Kashyap T, Dawson JL (2016). XPO1 inhibitor combination therapy with bortezomib or carfilzomib induces nuclear localization of IκBα and overcomes acquired proteasome inhibitor resistance in human multiple myeloma. Oncotarget.

[56] Gasparetto C, Schiller GJ, Tuchman SA (2022). Once weekly selinexor, carfilzomib and dexamethasone in carfilzomib non-refractory multiple myeloma patients. Br J Cancer.

[57] Bahlis NJ, Sutherland H, White D (2018). Selinexor plus low-dose bortezomib and dexamethasone for patients with relapsed or refractory multiple myeloma. Blood.

[58] Grosicki S, Simonova M, Spicka I (2020). Once-per-week selinexor, bortezomib, and dexamethasone versus twice-per-week bortezomib and dexamethasone in patients with multiple myeloma (BOSTON): a randomised, open-label, phase 3 trial. Lancet.

[59] Crochiere M, Kashyap T, Kalid O (2015). Deciphering mechanisms of drug sensitivity and resistance to selective inhibitor of nuclear export (SINE) compounds. BMC Cancer.

[60] Jardin F, Pujals A, Pelletier L (2016). Recurrent mutations of the exportin 1 gene (XPO1) and their impact on selective inhibitor of nuclear export compounds sensitivity in primary mediastinal B-cell lymphoma. Am J Hematol.

[61] Walker JS, Hing ZA, Harrington B (2021). Recurrent XPO1 mutations alter pathogenesis of chronic lymphocytic leukemia. J Hematol Oncol.

[62] Neggers JE, Vercruysse T, Jacquemyn M (2015). Identifying drug-target selectivity of small-molecule CRM1/XPO1 inhibitors by CRISPR/Cas9 genome editing. Chem Biol.

[63] Neggers JE, Vanstreels E, Baloglu E, Shacham S, Landesman Y, Daelemans D (2016). Heterozygous mutation of cysteine528 in XPO1 is sufficient for resistance to selective inhibitors of nuclear export. Oncotarget.

[64] Kashyap T, Argueta C, Aboukameel A (2016). Selinexor, a selective inhibitor of nuclear export (SINE) compound, acts through NF-κB deactivation and combines with proteasome inhibitors to synergistically induce tumor cell death. Oncotarget.

[65] Miyake TM, Pradeep S, Bayraktar E (2020). NRG1/ERBB3 pathway activation induces acquired resistance to XPO1 inhibitors. Mol Cancer Ther.

[66] Sun Z, Cui Y, Li Y, Li J, Qu X (2023). Dynamic single-cell RNA-seq reveals mechanism of selinexor-resistance in chronic myeloid leukemia. Blood.

[67] Lagana A, Park S, Edwards D (2018). E2F1 is a biomarker of selinexor resistance in relapsed/refractory multiple myeloma patients. Blood.

[68] Lagana A, Bhalla S, Aleman A (2019). A machine learning approach identifies a 30-gene model that predicts sensitivity to selinexor in multiple myeloma. Blood.

[69] Chari A, Cho HJ, Dhadwal A (2017). A phase 2 study of panobinostat with lenalidomide and weekly dexamethasone in myeloma. Blood Adv.

[70] Cho HJ, Mei AH, Tung K (2018). MAGE-A3 promotes chemotherapy resistance and proliferation in multiple myeloma through regulation of BIM and p21Cip1. Blood.

[71] Restrepo P, Bhalla S, Ghodke-Puranik Y (2022). A three-gene signature predicts response to selinexor in multiple myeloma. JCO Precis Oncol.

[72] Cohen YC, Zada M, Wang S (2021). Single cell RNA sequencing in patients enrolled in a selinexor clinical trial reveals overexpression of alternative nuclear export pathways associated with resistance to selinexor in refractory multiple myeloma. Blood.

[73] Wang X, Xu J, Li Q (2024). RNA-binding protein hnRNPU regulates multiple myeloma resistance to selinexor. Cancer Lett.

[74] Krecic AM, Swanson MS (1999). hnRNP complexes: composition, structure, and function. Curr Opin Cell Biol.

[75] Zhu ZC, Liu JW, Yang C, Zhao M, Xiong ZQ (2019). XPO1 inhibitor KPT-330 synergizes with Bcl-xL inhibitor to induce cancer cell apoptosis by perturbing rRNA processing and Mcl-1 protein synthesis. Cell Death Dis.

[76] Hu F, Chen XQ, Li XP (2022). Drug resistance biomarker ABCC4 of selinexor and immune feature in multiple myeloma. Int Immunopharmacol.

[77] Li S, Fu J, Walker CJ (2023). Dual targeting of protein translation and nuclear protein export results in enhanced antimyeloma effects. Blood Adv.

[78] Liao Y, Ke X, Deng T, Qin Q (2021). The second-generation XPO1 inhibitor eltanexor inhibits human cytomegalovirus (HCMV) replication and promotes type I interferon response. Front Microbiol.

[79] Lancman G, Sastow DL, Cho HJ (2021). Bispecific antibodies in multiple myeloma: present and future. Blood Cancer Discov.

[80] Kontermann RE, Brinkmann U (2015). Bispecific antibodies. Drug Discov Today.

[81] Rodríguez-Lobato LG, Ganzetti M, Fernández de Larrea C, Hudecek M, Einsele H, Danhof S (2020). CAR T-cells in multiple myeloma: state of the art and future directions. Front Oncol.

[82] Martin T, Usmani SZ, Berdeja JG (2023). Ciltacabtagene autoleucel, an anti-B-cell maturation antigen chimeric antigen receptor T-cell therapy, for relapsed/refractory multiple myeloma: CARTITUDE-1 2-year follow-up. J Clin Oncol.

[83] Rodríguez Otero P, Ailawadhi S, Arnulf B (2023). Idecabtagene vicleucel (ide-cel) versus standard (std) regimens in patients (pts) with triple-class-exposed (TCE) relapsed and refractory multiple myeloma (RRMM): updated analysis from KarMMa-3. Blood.

[84] Ali SA, Shi V, Maric I (2016). T cells expressing an anti-B-cell maturation antigen chimeric antigen receptor cause remissions of multiple myeloma. Blood.

[85] Brudno JN, Maric I, Hartman SD (2018). T cells genetically modified to express an anti-B-cell maturation antigen chimeric antigen receptor cause remissions of poor-prognosis relapsed multiple myeloma. J Clin Oncol.

[86] Samur MK, Fulciniti M, Aktas Samur A (2021). Biallelic loss of BCMA as a resistance mechanism to CAR T cell therapy in a patient with multiple myeloma. Nat Commun.

[87] Da Vià MC, Dietrich O, Truger M (2021). Homozygous BCMA gene deletion in response to anti-BCMA CAR T cells in a patient with multiple myeloma. Nat Med.

[88] Mailankody S, Devlin SM, Landa J (2022). GPRC5D-targeted CAR T cells for myeloma. N Engl J Med.

[89] Brioli A, Melchor L, Cavo M, Morgan GJ (2014). The impact of intra-clonal heterogeneity on the treatment of multiple myeloma. Br J Haematol.

[90] Eckmann J, Hage C, Stefanie L, Bayer C, Klein C, Umana P (2023). Early intervention with celmods, but not imids, prevents relapse to forimtamig driven by GPRC5D-negative myeloma cells. Blood.

[91] Hamieh M, Dobrin A, Cabriolu A (2019). CAR T cell trogocytosis and cooperative killing regulate tumour antigen escape. Nature.

[92] (2021). van de Donk NWCJ, Themeli M, Usmani SZ. Determinants of response and mechanisms of resistance of CAR T-cell therapy in multiple myeloma. Blood Cancer Discov.

[93] Long AH, Haso WM, Shern JF (2015). 4-1BB costimulation ameliorates T cell exhaustion induced by tonic signaling of chimeric antigen receptors. Nat Med.

[94] Meermeier EW, Welsh SJ, Sharik ME (2021). Tumor burden limits bispecific antibody efficacy through T cell exhaustion averted by concurrent cytotoxic therapy. Blood Cancer Discov.

[95] Philipp N, Kazerani M, Nicholls A (2022). T-cell exhaustion induced by continuous bispecific molecule exposure is ameliorated by treatment-free intervals. Blood.

[96] Cohen AD, Mateos MV, Cohen YC (2023). Efficacy and safety of cilta-cel in patients with progressive multiple myeloma after exposure to other BCMA-targeting agents. Blood.

[97] Lee H, Neri P, Bahlis NJ (2023). Current use of bispecific antibodies to treat multiple myeloma. Hematology Am Soc Hematol Educ Program.

[98] Cortes-selva D, Casneuf T, Vishwamitra D (2022). Teclistamab, a B-cell maturation antigen (BCMA) × CD3 bispecific antibody, in patients with relapsed/refractory multiple myeloma (RRMM): correlative analyses from majesTEC-1. Blood.

[99] Ahn S, Leblay N, Neri P (2021). Understanding the mechanisms of resistance to T cell-based immunotherapies to develop more favorable strategies in multiple myeloma. Hemasphere.

[100] Leblay N, Maity R, Hasan F, Neri P (2020). Deregulation of adaptive T cell immunity in multiple myeloma: insights into mechanisms and therapeutic opportunities. Front Oncol.

[101] Vishwamitra D, Skerget S, Cortes D (2023). Mechanisms of resistance and relapse with talquetamab in patients with relapsed/refractory multiple myeloma from the phase 1/2 monumenTAL-1 study. Blood.

[102] Friedrich MJ, Neri P, Kehl N (2023). The pre-existing T cell landscape determines the response to bispecific T cell engagers in multiple myeloma patients. Cancer Cell.

[103] Thakurta A, Pierceall WE, Amatangelo MD, Flynt E, Agarwal A (2021). Developing next generation immunomodulatory drugs and their combinations in multiple myeloma. Oncotarget.

[104] Ruchelman AL, Man HW, Zhang W (2013). Isosteric analogs of lenalidomide and pomalidomide: synthesis and biological activity. Bioorg Med Chem Lett.

[105] Chamberlain PP, Lopez-Girona A, Miller K (2014). Structure of the human cereblon-DDB1-lenalidomide complex reveals basis for responsiveness to thalidomide analogs. Nat Struct Mol Biol.

[106] Matyskiela ME, Zhang W, Man HW (2018). A cereblon modulator (CC-220) with improved degradation of ikaros and aiolos. J Med Chem.

[107] Hansen JD, Correa M, Nagy MA (2020). Discovery of CRBN E3 ligase modulator CC-92480 for the treatment of relapsed and refractory multiple myeloma. J Med Chem.

[108] Bjorklund CC, Kang J, Amatangelo M (2020). Iberdomide (CC-220) is a potent cereblon E3 ligase modulator with antitumor and immunostimulatory activities in lenalidomide- and pomalidomide-resistant multiple myeloma cells with dysregulated CRBN. Leukemia.

[109] Amatangelo M, Bjorklund C, Ma P (2020). Preclinical and translational data support development of iberdomide in combination with CD38- and SLAMF7-directed monoclonal antibodies: evidence for rational combinations. Blood.

[110] Van Oekelen O, Amatangelo M, Guo M (2021). Large-scale mass cytometry reveals significant activation of innate and adaptive immunity in bone marrow tumor microenvironment of iberdomide-treated myeloma patients. Blood.

[111] Ma P, Sridharan V, Wollerman K (2023). Iberdomide enhances dara mediated cytotoxicity through upregulation of CDC activity and elevated NK cell mediated ADCC. Blood.

[112] (2020). van de Donk NWCJ, Popat R, Larsen J, et al. First results of iberdomide (IBER; CC-220) in combination with dexamethasone (DEX) and daratumumab (DARA) or bortezomib (BORT) in patients with relapsed/refractory multiple myeloma (RRMM). Blood.

[113] van de Donk NW, Touzeau C, Terpos E (2023). Iberdomide maintenance after autologous stem-cell transplantation in newly diagnosed MM: first results of the phase 2 EMN26 study. Blood.

[114] Lonial S, Abdallah A, Anwer F (2022). Iberdomide (IBER) in combination with dexamethasone (DEX) in relapsed/refractory multiple myeloma (RRMM): results from the anti-B-cell maturation antigen (BCMA)-exposed cohort of the CC-220-MM-001 trial. Blood.

[115] Richardson PG, Trudel S, Quach H (2022). Mezigdomide (CC-92480), a potent, novel cereblon E3 ligase modulator (CELMoD), combined with dexamethasone (DEX) in patients (pts) with relapsed/refractory multiple myeloma (RRMM): preliminary results from the dose-expansion phase of the CC-92480-MM-001 trial. Blood.

[116] Richardson PG, Sandhu I, Hofmeister CC (2023). Mezigdomide (MEZI) plus dexamethasone (DEX) and daratumumab (DARA) or elotuzumab (ELO) in patients (pts) with relapsed/refractory multiple myeloma (RRMM): results from the CC-92480-MM-002 trial. Blood.

[117] Bird S, Pawlyn C (2023). IMiD resistance in multiple myeloma: current understanding of the underpinning biology and clinical impact. Blood.

[118] Bohl SR, Schmalbrock LK, Bauhuf I (2021). Comprehensive CRISPR-Cas9 screens identify genetic determinants of drug responsiveness in multiple myeloma. Blood Adv.

[119] Kortüm KM, Mai EK, Hanafiah NH (2016). Targeted sequencing of refractory myeloma reveals a high incidence of mutations in CRBN and Ras pathway genes. Blood.

[120] Gooding S, Ansari-Pour N, Towfic F (2021). Multiple cereblon genetic changes are associated with acquired resistance to lenalidomide or pomalidomide in multiple myeloma. Blood.

[121] Chrisochoidou Y, Lebihan Y, Morales S (2023). Investigating the functional impact of CRBN mutations on response to IMiD/celmod agents in myeloma. Blood.

[122] Tilmont R, Maity R, Leblay N (2022). CRBN structural changes, copy number changes and COP9 signalosome subunits gene expression mediate sensitivity to new celmod compound CC-92480 in multiple myeloma patients. Blood.

[123] Haertle L, Barrio S, Munawar U (2021). Cereblon enhancer methylation and IMiD resistance in multiple myeloma. Blood.

[124] Dimopoulos K, Søgaard Helbo A, Fibiger Munch-Petersen H (2018). Dual inhibition of DNMTs and EZH2 can overcome both intrinsic and acquired resistance of myeloma cells to IMiDs in a cereblon-independent manner. Mol Oncol.

[125] Bird SA, Barber A, Sialana FJ (2022). Multiomics analysis of IMiD/CELMoD resistant multiple myeloma models uncovers novel and targetable vulnerabilities in the SREBP lipid synthesis pathway. Blood.

[126] Barrio S, Munawar U, Zhu YX (2020). IKZF1/3 and CRL4^CRBN^ E3 ubiquitin ligase mutations and resistance to immunomodulatory drugs in multiple myeloma. Haematologica.

[127] Chung DJ, Pronschinske KB, Shyer JA (2016). T-cell exhaustion in multiple myeloma relapse after autotransplant: optimal timing of immunotherapy. Cancer Immunol Res.

[128] Batorov EV, Aristova TA, Sergeevicheva VV (2020). Quantitative and functional characteristics of circulating and bone marrow PD-1- and TIM-3-positive T cells in treated multiple myeloma patients. Sci Rep.

[129] Lucas F, Pennell M, Huang Y (2020). T cell transcriptional profiling and immunophenotyping uncover LAG3 as a potential significant target of immune modulation in multiple myeloma. Biol Blood Marrow Transplant.

[130] Chen LY, Gooding S (2022). Tumor and microenvironmental mechanisms of resistance to immunomodulatory drugs in multiple myeloma. Front Oncol.

[131] Biran N, Vesole DH, Parmar H (2023). A phase 2b study of selinexor in combination with carfilzomib, daratumumab, or pomalidomide in patients with multiple myeloma relapsing on current therapy. Blood.

[132] Gasparetto C, Lentzsch S, Schiller G (2021). Selinexor, daratumumab, and dexamethasone in patients with relapsed or refractory multiple myeloma. EJHaem.

[133] White DJ, Chen CI, Baljevic M (2021). Once weekly oral selinexor, pomalidomide, and dexamethasone in relapsed refractory multiple myeloma. Blood.

[134] White D, Schiller GJ, Madan S (2024). Efficacy and safety of once weekly selinexor 40 mg versus 60 mg with pomalidomide and dexamethasone in relapsed and/or refractory multiple myeloma. Front Oncol.

[135] Mei H, Li C, Jiang H (2021). A bispecific CAR-T cell therapy targeting BCMA and CD38 in relapsed or refractory multiple myeloma. J Hematol Oncol.

[136] Tang Y, Yin H, Zhao X (2022). High efficacy and safety of CD38 and BCMA bispecific CAR-T in relapsed or refractory multiple myeloma. J Exp Clin Cancer Res.

[137] Yan Z, Cao J, Cheng H (2019). A combination of humanised anti-CD19 and anti-BCMA CAR T cells in patients with relapsed or refractory multiple myeloma: a single-arm, phase 2 trial. Lancet Haematol.

[138] Zhang X, Zhang H, Lan H, Wu J, Xiao Y (2023). CAR-T cell therapy in multiple myeloma: current limitations and potential strategies. Front Immunol.

[139] Zhang X, Zhang C, Qiao M (2022). Depletion of BATF in CAR-T cells enhances antitumor activity by inducing resistance against exhaustion and formation of central memory cells. Cancer Cell.

[140] Zhang H, Hu Y, Shao M (2021). Dasatinib enhances anti-leukemia efficacy of chimeric antigen receptor T cells by inhibiting cell differentiation and exhaustion. J Hematol Oncol.

[141] Ali AI, Wang M, von Scheidt B (2021). A histone deacetylase inhibitor, panobinostat, enhances chimeric antigen receptor T-cell antitumor effect against pancreatic cancer. Clin Cancer Res.

[142] Lesokhin AM, Richter J, Trudel S (2022). Enduring responses after 1-year, fixed-duration cevostamab therapy in patients with relapsed/refractory multiple myeloma: early experience from a phase I study. Blood.

[143] Abecassis A, Roders N, Fayon M (2022). CAR-T cells derived from multiple myeloma patients at diagnosis have improved cytotoxic functions compared to those produced at relapse or following daratumumab treatment. EJHaem.

[144] Searle E, Quach H, Wong SW (2022). Teclistamab in combination with subcutaneous daratumumab and lenalidomide in patients with multiple myeloma: results from one cohort of majesTEC-2, a phase1b, multicohort study. Blood.

[145] Cohen YC, Morillo D, Gatt ME (2023). First results from the RedirecTT-1 study with teclistamab (tec) + talquetamab (tal) simultaneously targeting BCMA and GPRC5D in patients (pts) with relapsed/refractory multiple myeloma (RRMM). J Clin Oncol.

[146] Mateos M, Morillo D, Gatt M (2023). S190: First results from the redirectt-1 study with teclistamab (TEC) + talquetamab (TAL) simultaneously targeting bcma and GPRC5D in patients (PTS) with relapsed/refractory multiple myeloma (RRMM). HemaSphere.

[147] Pillarisetti R, Yang D, Yao J (2023). Characterization of JNJ-79635322, a novel BCMAxGPRC5DxCD3 T-cell redirecting trispecific antibody, for the treatment of multiple myeloma. Blood.

[148] Lee H, Neri P, Bahlis NJ (2024). BCMA- or GPRC5D-targeting bispecific antibodies in multiple myeloma: efficacy, safety, and resistance mechanisms. Blood.

[149] Chow TT, Amatangelo M, Ma P (2023). Preclinical and translational biomarker analyses to inform clinical development of mezigdomide (CC-92480) in combination with dexamethasone and daratumumab in multiple myeloma. Blood.

[150] Bjorklund CC, Amatangelo M, Kang J (2021). CC-92480 enhances cell-autonomous cytotoxicity through blockade of G 2/M transition when combined with bortezomib/dexamethasone in pre-clinical multiple myeloma. Blood.

[151] Amatangelo M, Bjorklund CC, Hagner P (2022). P-230: Preclinical and translational biomarker analysis to support further clinical development and dose optimization of mezigdomide (MEZI; CC-92480) in combination with either bortezomib or carfilzomib. Cl Lymph Myelom Leuk.

[152] Paiva B, Gaffney B, Burnett K (2022). Synergistic antitumor activity of alnuctamab (ALNUC; BMS-986349; CC-93269), a BCMA 2+1 T cell engager (TCE), and celmod agents in multiple myeloma (MM) preclinical models. Blood.

[153] Aleman A, Kogan-zajdman A, Upadhyaya B (2023). P-175 improving anti-BCMA CAR-T functionality with novel immunomodulatory agent iberdomide (CC220) in multiple myeloma. Cl Lymph Myelom Leuk.

[154] Rodriguez Otero P, D’souza A, Reece D (2022). S188: Teclistamab in combination with daratumumab, a novel, immunotherapy-based approach for the treatment of relapsed/refractory multiple myeloma: updated phase 1b results. HemaSphere.

[155] Matous J, Biran N, Perrot A (2023). Talquetamab + pomalidomide in patients with relapsed/refractory multiple myeloma: safety and preliminary efficacy results from the phase 1b monumenTAL-2 study. Blood.

[156] Dholaria BR, Weisel K, Mateos M (2023). Talquetamab (tal) + daratumumab (dara) in patients (pts) with relapsed/refractory multiple myeloma (RRMM): updated TRIMM-2 results. J Clin Oncol.

[157] Biran N, Vesole DH, Parmar H (2023). A phase 1/2 study of carfilzomib, iberdomide and dexamethasone (KID) in patients with newly diagnosed transplant-eligible multiple myeloma. Blood.

[158] Lonial S, Richardson PG, Popat R (2021). OAB-013: iberdomide (IBER) in combination with dexamethasone (DEX) and daratumumab (DARA), bortezomib (BORT), or carfilzomib (CFZ) in patients (pts) with relapsed/refractory multiple myeloma (RRMM). Cl Lymph Myelom Leuk.

[159] White D, Lipe B, Mesa MG (2023). OA-41 Iberdomide, bortezomib, and dexamethasone (IberVd) in transplant-ineligible newly diagnosed multiple myeloma (NDMM): results from the CC-220-MM-001 trial. Cl Lymph Myelom Leuk.

[160] Oriol A, Sandhu I, Raab M (2023). OA-49 Mezigdomide (MEZI) plus dexamethasone (DEX) and bortezomib (BORT) or carfilzomib (CFZ) in patients (pts) with relapsed/refractory multiple myeloma (RRMM): results from the CC-92480-MM-002 trial. Cl Lymph Myelom Leuk.

